# Poly(ADP-ribose)polymerases inhibitors prevent early mitochondrial fragmentation and hepatocyte cell death induced by H_2_O_2_

**DOI:** 10.1371/journal.pone.0187130

**Published:** 2017-10-26

**Authors:** Sandra M. Martín-Guerrero, José A. Muñoz-Gámez, María-Carmen Carrasco, Javier Salmerón, María Martín-Estebané, Miguel A. Cuadros, Julio Navascués, David Martín-Oliva

**Affiliations:** 1 Departamento de Biología Celular, Facultad de Ciencias, Universidad de Granada, Granada, Spain; 2 Instituto de Investigación Biomédica (ibsGranada), Hospital Universitario San Cecilio, Granada, Spain; University of South Alabama Mitchell Cancer Institute, UNITED STATES

## Abstract

Poly(ADP-ribose)polymerases (PARPs) are a family of NAD^+^ consuming enzymes that play a crucial role in many cellular processes, most clearly in maintaining genome integrity. Here, we present an extensive analysis of the alteration of mitochondrial morphology and the relationship to PARPs activity after oxidative stress using an *in vitro* model of human hepatic cells. The following outcomes were observed: reactive oxygen species (ROS) induced by oxidative treatment quickly stimulated PARPs activation, promoted changes in mitochondrial morphology associated with early mitochondrial fragmentation and energy dysfunction and finally triggered apoptotic cell death. Pharmacological treatment with specific PARP-1 (the major NAD^+^ consuming poly(ADP-ribose)polymerases) and PARP-1/PARP-2 inhibitors after the oxidant insult recovered normal mitochondrial morphology and, hence, increased the viability of human hepatic cells. As the PARP-1 and PARP-1/PARP-2 inhibitors achieved similar outcomes, we conclude that most of the PARPs effects were due to PARP-1 activation. NAD^+^ supplementation had similar effects to those of the PARPs inhibitors. Therefore, PARPs activation and the subsequent NAD^+^ depletion are crucial events in decreased cell survival (and increased apoptosis) in hepatic cells subjected to oxidative stress. These results suggest that the alterations in mitochondrial morphology and function seem to be related to NAD^+^ depletion, and show for the first time that PARPs inhibition abrogates mitochondrial fragmentation. In conclusion, the inhibition of PARPs may be a valuable therapeutic approach for treating liver diseases, by reducing the cell death associated with oxidative stress.

## Introduction

The liver is a vital organ that plays a decisive role in detoxification, and therefore hepatic damage is frequently the cause of severe pathologies. One of the main factors provoking hepatocyte degeneration (and consequently liver damage) is oxidative stress, which is often associated with the detoxification function of the liver. Oxidative stress in a cell develops when there is an imbalance between the amount of reactive oxygen species (ROS) present and the ability of the cell of eliminate it or to repair the damage resulting from the action of ROS. Oxidative stress leads to multiple types of cell damage, including DNA breaks, protein modifications, lipid peroxidation, disruption of calcium homeostasis, mitochondrial failure, impairment of the energy metabolism and NAD^+^ depletion [1]. Oxidative stress is apparently at the origin of most liver diseases, such as those caused by alcohol consumption [2, 3], hepatotoxic drugs [4, 5], environmental pollutants [6] and other factors [7, 8]. Moreover, oxidative stress in patients suffering non-alcoholic fatty liver disease (NAFLD) is significantly greater than in healthy controls [9].

ROS is a collective term that includes oxygen free radicals (such as superoxide anion, hydroxyl and hydroperoxyl radical) and nonradical agents (such as hydrogen peroxide [H_2_O_2_], singlet oxygen and peroxynitrite) with oxidising capacity [10]. ROS are produced as a consequence of oxidative processes that take place in various types of membrane organelles, especially in the mitochondria during aerobic metabolism [11]. As well as being crucial for energy production, the mitochondria also participate in other aspects of cell activity such as apoptosis and the biosynthesis of certain molecules. Consequently, when ROS are present in excess, affecting mitochondrial function, they may originate bioenergetic and metabolic flaws that underlie a heterogeneous group of human diseases [12, 13]. In particular, mitochondrial dysfunction and oxidative stress have been documented in the progression of various liver diseases, including NAFLD and non-alcoholic steatohepatitis [14, 15].

Mitochondria are dynamic organelles that present different morphologies, ranging from small spherical forms to long interconnected tubular networks, depending on the cell type and the physiological conditions. In fact, these morphologies may be affected by the alteration of mitochondrial function. This behaviour is generated by mitochondrial fission, which fragments the mitochondria, producing small spherical forms, and also by fusion processes, which give rise to tubular networks of interconnected mitochondria [16, 17]. Increased ROS levels induce mitochondrial fragmentation in different cell lines [16], an outcome that seems to be a hallmark of apoptosis; it is widespread in apoptotic cell death and blocking mitochondrial fission delays apoptosis [18].

Poly(ADP-ribose)polymerases (PARPs) are a family of nuclear proteins that consume NAD^+^ to modify target proteins. In humans, PARPs family members are encoded by 17 genes and the main member of this family is the enzyme poly(ADP-ribose)polymerase-1 (PARP-1), which is responsible for 85–90% of PARPs activity (and NAD^+^ consumption), followed by PARP-2, which accounts for the remaining 10–15% of activity [19]. Only these two members of the PARPs family have been implicated in DNA damage sensing and repairing [20]. Thus, ROS exposure, inducing strand breaks in DNA, increases the activation of PARP-1 and PARP-2 (hereafter referred to, jointly, as PARPs). Activated PARPs cleave NAD^+^ substrates to form poly-ADP-ribose (PAR) polymers that are added to themselves and to other protein acceptors. Many PAR-modified proteins are related to DNA repair [21]. Overactivation of PARPs depletes the NAD^+^ from the cell and consequently induces a dramatic reduction in ATP levels [22]. Thus, extensive PARPs activation could result in a global energy failure, causing necrosis [23], which is implicated in many inflammatory diseases [24, 25].

In the present study, an *in vitro* model of human hepatic cells exposed for short times to high levels of a pro-oxidant agent was used to determine whether PARPs inhibition exerts a protective role against mitochondrial dysfunction and cell death. Our results show that PARPs inhibitors increase the viability of hepatocytes and enable the recovery of normal mitochondrial morphology after the oxidant insult. Thus, PARPs inhibition could be a novel target to suppress both the cell death associated with oxidative stress and the pathological outcome of related hepatic diseases.

## Materials and methods

### Cell culture and treatments

WRL68 human liver cells (hepatic cells) were purchased by the Scientific Instrumentation Services (CIC) of the University of Granada from the European Collection of Cell Cultures (cat # 89121403). Hepatic cells were maintained as an adherent cultured monolayer in DMEM (Sigma, St. Louis, MO) added with 10% foetal bovine serum (Sigma), L-glutamine solution (2 mM; Sigma) containing streptomycin (100 μg/ml) and penicillin (100 U/ml), and incubated at 37°C in 5% CO_2_.

**Fig 1:**
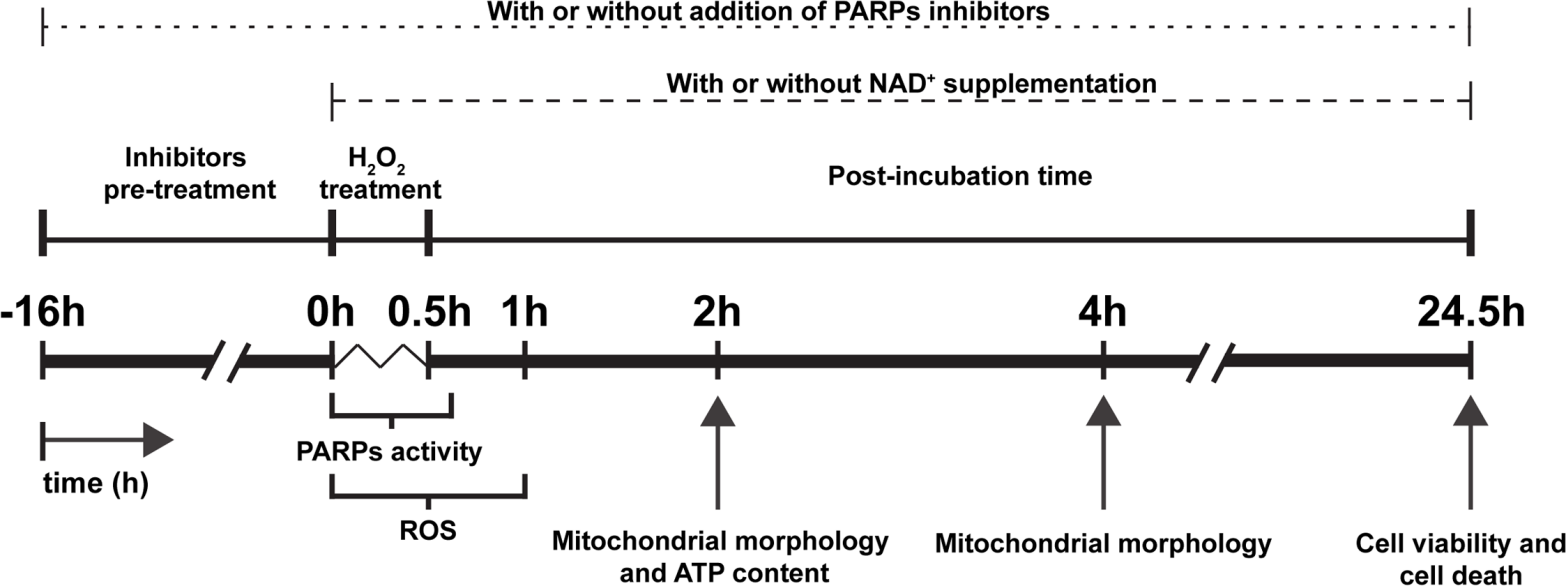
Schematic summary of experimental procedures. Oxidative treatment was applied as a single dose of H_2_O_2_ for 30 min. PARPs activity was analysed during oxidative treatment and 15 minutes afterwards. ROS production was analysed during H_2_O_2_ treatment and after 30 min of post-incubation. Cell viability and characterisation of cell death were determined after 24 h of post-incubation. Mitochondrial morphology was studied at 2 h or 4 h after H_2_O_2_ exposure. Cellular ATP content was studied at 2 h after the end of the H_2_O_2_ treatment. PARPs inhibitors were added to culture media 16 h before oxidative treatment, during the treatment and during post-incubation. Media were supplemented with NAD^+^ during H_2_O_2_ treatment and during post-incubation.

In order to induce ROS production and oxidative insult, hepatic cell cultures were treated with a single dose of H_2_O_2_ (from a 30% stock solution, Sigma) diluted to concentrations ranging from 0.25 to 5 mM for 0–30 min. The medium was then replaced with fresh growth medium, and the cells were further incubated for 2, 4 or 24 h depending on the experiment (Fig 1).

PJ34 (Enzo Life Sciences, San Diego, CA) and AG14361 (Selleck Chemicals, Houston, TX) were used as PARPs inhibitors. PJ34 is a water-soluble cell-permeable phenanthridinone derivative which selectively inhibits the catalytic activity of PARP-1 and PARP-2 (EC_50_ = 20 nM) [26]; while AG14361, a tricyclic benzimidazole, is an extremely potent inhibitor specific for PARP-1 (K_i_ <5 nM) [27]. The cells were pre-incubated (this is termed the Inhibitor pre-treatment step in Fig 1) with the PJ34 or AG14361 at a concentration of 1 μM for 16 h before the oxidativetreatment; the same PARPs inhibitor and concentration were also present in the medium of H_2_O_2_ incubation and in the fresh medium added post-treatment (termed the Post-incubation time step in Fig 1).

To avoid NAD^+^ depletion, exogenous NAD^+^ (0.25 mM, Sigma) was added to the cell culture during the H_2_O_2_ treatment and to the fresh medium during post-treatment. Some cell cultures were treated with 4 mM of sodium pyruvate (Sigma), a ROS scavenger, during the H_2_O_2_ treatment and post-treatment.

### Measurements of intracellular ROS

The hepatic cells were seeded in 6-well culture plates at a density of 2.0 x 10^5^ cells/well. Before the H_2_O_2_ treatment, the cells were incubated with 20 μM of dichlorodihydrofluorescein diacetate (H_2_DCFDA) for 30 min. This probe is cell-permeable and is hydrolysed intracellularly to the DCFH carboxylate anion, which is retained in the cell. Oxidation of DCFH by intracellular ROS results in the formation of the fluorescent product dichlorofluorescein (DCF) [10]. After H_2_DCFDA incubation, the medium was removed and the cells were treated with H_2_O_2_ for 0–30 min (Fig 1). The cells were then detached with trypsin-EDTA solution (Sigma), centrifuged at 300 g and washed with ice-cold PBS. Finally, the fluorescent staining was detected by flow cytometry analysis in a Becton Dickinson FACSAria III cytometer using FACSDiva software (BD Biosciences, Erembodegem, Belgium). The number of fluorescent positive-cells was expressed as a percentage of the total number of cells. It is important to note that DCFH does not directly react with H_2_O_2_ to form the fluorescent product DCF [10].

### Immunofluorescence and PARPs activity

Hepatic cells seeded on coverslips in 6-well culture plates were treated for 0–30 min with H_2_O_2_ and fixed within 15 min with ice-cold methanol-acetone 1:1 for 10 min to determine the time-course of maximum activity of PARPs (Fig 1). The coverslips were incubated overnight at 4°C with the primary mouse monoclonal anti-PAR antibody. Two antibodies were indistinctly used for these immunofluorescence studies: cat # 4335-MC-100 (Trevigen, Gaithersburg, MD) and cat # ALX-804-220-R100 (Enzo Life Sciences, San Diego, CA) [28], both at a dilution of 1:400. Then, the coverslips were incubated with the secondary antibody (Alexa fluor 488-conjugated goat anti-mouse IgG; Molecular Probes, Eugene, OR; cat # A-11001; dilution 1:1000) at room temperature for 2 h and cell nuclei were counterstained with the dye Hoechst 33342 (Sigma) for 2 min. Finally, the coverslips were mounted on slides using Fluoromount G mounting medium (Southern Biotech, Birmingham, AL), and analysed using an Axiophot microscope (Zeiss, Oberkochen, Germany). To determine the level of PARPs activation, the number of PAR-positive nuclei detected at 400X magnification was counted in a total of 200 nuclei for each condition, from three separate experiments.

### Determination of cell viability and cell death

Cell viability was determined using the MTT (3-[4,5-dimethylthiazol-2-yl]-2,5-diphenyl tetrazolium bromide, Sigma) method [29]. The cells were seeded in 96-well culture plates at an initial density of 8.0 x 10^3^ cells/well. After 30 min of H_2_O_2_ treatment, the cells were incubated for 24 h (post-incubation time) in fresh medium for recovery, and 5 mg/ml MTT was then added for 3 h to measure cell viability (Fig 1). After this, the culture medium was removed and 100 μl/well of DMSO (Sigma) were added to dissolve the formazan crystals. The absorbance of each well at 595 nm was measured in a microplate spectrophotometer reader (Multiskan Ascent, Thermo Scientific, Rockford, lL). Cell viability in each well was expressed as a percentage of the absorbance for each experimental condition in relation to the control wells (untreated cells), assuming that untreated cells represented 100% viability.

Apoptotic cell death was measured using an Annexin-V-FITC kit with propidium iodide solution (InmunoStep, Salamanca, Spain; cat # ANXVF-200T) to detect phosphatidylserine exposure [30]. Hepatic cells, seeded in 6-well culture plates, were treated for 30 min with H_2_O_2_, and after 24 h of post-incubation time (Fig 1) were detached, labelled following the manufacturer’s protocol and analysed with a Becton Dickinson FACSAria III cytometer using FACSDiva software (BD Biosciences). Cells treated with 6 μM of staurosporine (Sigma) for 24 h served as positive controls for apoptosis [31].

Necrotic cell death was evaluated by examining the release of lactate dehydrogenase (LDH) [32]. In this method, the cells were seeded in 96-well culture plates at an initial density of 12.0 x 10^3^ cells/well and cultured overnight at 37°C before H_2_O_2_ treatment (Fig 1); after 24 h of post-incubation, LDH release was assessed by measuring LDH activity in culture medium using a LDH Cytotoxicity Assay Kit (Thermo Scientific; cat # 88953) according to the manufacturer’s instructions. The absorbance (Abs) at 490 nm of cell-culture medium from each experimental condition (Abs exp) was measured in the microplate spectrophotometer reader (Multiskan Ascent, Thermo Scientific); to determine the Abs variation due to the experimental conditions, the background Abs values were subtracted in each Abs exp. Some cultures were treated with 1% Triton (Sigma) for 10 min as a positive control for necrosis: absorbance corresponding to the spontaneous LDH release (Abs spont) was determined in untreated cells, while that of the Triton-treated cells corresponded to the maximum LDH release (Abs max). The percentages (%) of LDH release were determined as *LDH release (%) = (Abs exp—Abs spont) / (Abs max–Abs spont)*.

### Mitochondrial morphology quantification and ultrastructural study

Changes in mitochondrial morphology and ultrastructure were studied in hepatic cells seeded on coverslips in 6-well culture plates subjected to 30 min of hydrogen peroxide treatment, and post-incubated in fresh medium for 2 or 4 h (Fig 1).

Two strategies were employed in this study: first, the fluorescent mitochondrial probe Mitotracker Red CMXROS (Cell Signaling Technology, Danvers, MA) was added at a concentration of 125 nM to the cells during the last 45 min of post-incubation. Then, the cells were fixed with ice-cold methanol-acetone 1:1 for 10 min, mounted on slides using Fluoromount G (SouthernBiotech), and analysed with a confocal Leica TCS-SP microscope (Leica, Wetzlar, Germany). The mitochondria were analysed and classified using MicroP software, a useful tool that has been used previously to study mitochondrial fragmentation [33]. Two major types of mitochondria were considered, in terms of the morphology observed: a) Type I, small mitochondria with globular morphology; b) Type II, mitochondria with tubular morphologies (including branched, twisted or straight tubules). Approximately 7,000 mitochondria were analysed in 10 high-power fields per condition in each separate experiment at 600x magnification. The elongation index and the mitochondrialarea were also measured with MicroP software. The elongation index was calculated as the length of the mitochondria divided by their width. A larger elongation index corresponded to tubular mitochondria morphology.

In the second strategy, the cells were processed for transmission electron microscopy (TEM) by fixing them in 2% glutaraldehyde in 0.05 M cacodylate buffer (pH 7.4) supplemented with 2 mM Cl_2_Mg and 0.03 g/L sucrose for 2 h, postfixed in 1% osmium tetroxide for 1 h, dehydrated in graded series of ethanol, and embedded in epoxy resin. Ultrathin sections (50–70 nm) were mounted on copper grids and examined under a Zeiss Libra 120 EDX electron microscope (Zeiss, Oberkochen, Germany).

### Cellular ATP assay

WRL68 cells were seeded in a 25 cm^2^ flask at a density of 1.0 x 10^6^ cells/flask and allowed to grow for 24 h. The cells were then treated with H_2_O_2_ for 30 min and post-incubated for 2 h in fresh medium (Fig 1). After this, the cells were recovered, centrifuged at 300 g for 10 min and washed with PBS. Intracellular ATP was extracted using the boiling water procedure described by Yang and coworkers [34]: 400 μl of boiling water was added to each cellular pellet, which was subsequently vortexed and incubated for 5 min; the resulting suspension was centrifuged at 12000 g for 5 min. ATP content in the supernatant was determined using the Adenosine 5’-triphosphate (ATP) Bioluminescent Assay Kit (Sigma, cat # FLAA-1KT) according to the manufacturer’s instructions. Luminescence was measured from 96-well plates using a microplate reader (TRIAD series multimode detector, Dynex Technologies, Chantilly, VA) and the quantity of ATP was calculated using a standard curve. Proteins were measured using the Bradford method (Biorad). ATP content was expressed as nmol per mg of total protein.

### Statistical analysis

Data are expressed as mean ± SEM from at least three independent experiments. Student’s t-test was used to determine significant differences. Statistical analyses were performed using IBM-SPSS Statistics version 19.0 software (IBM Corp., Armonk, NY). A value of *P* < 0.05 was considered statistically significant.

## Results

### A single short exposure to an oxidizing agent reduce hepatic cell viability

**Fig 2:**
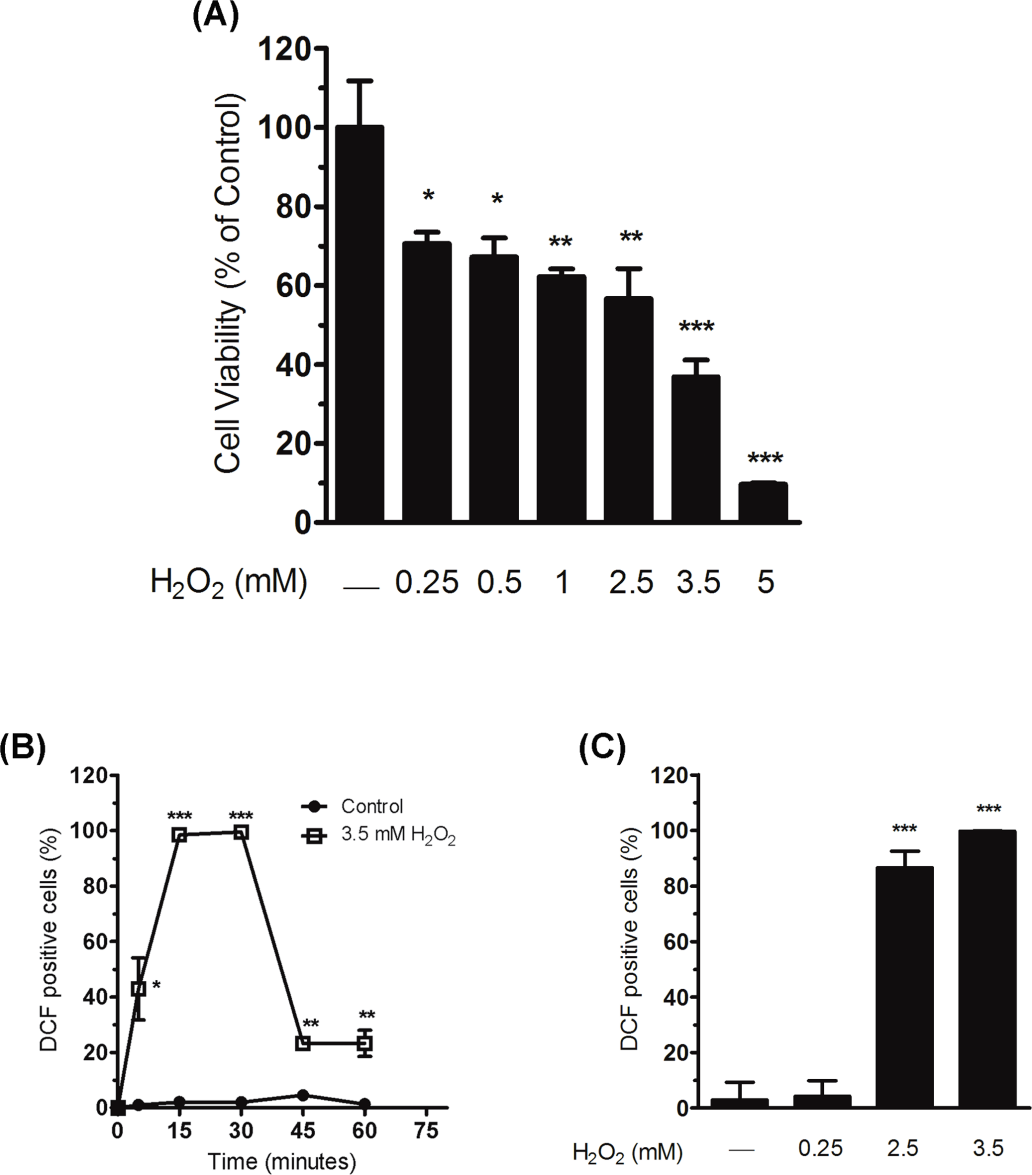
Decreased cell viability and ROS production induced by oxidative treatment. (A) Twenty-four hours after exposure to diverse concentrations of H_2_O_2_ for 30 min, the viability of WRL68 cells was analysed with the MTT assay. Cell viability was expressed as the percentage of viable cells with respect to those determined in the controls (H_2_O_2_ non-treated cells), which was considered to be 100%. Bar graph shows the mean ± SEM of three independent experiments. Significant differences with respect to the controls (H_2_O_2_ non-treated cells): *P < 0.05, **P < 0.01 and ***P < 0.001. (B) ROS production by WRL68 cells was evaluated by incubating the cells with H_2_DCFDA for 30 min, before exposing them to H_2_O_2_. The level of DCF fluorescence was evaluated by flow cytometry at 0, 5, 15 and 30 min during exposure and at 15 and 30 min after 30 min exposure to H_2_O_2_ (time point of 45 min and 60 min, respectively, in the figure). The linear graph shows the percentage of fluorescent cells (DCF positive cells) at each time point. Mean ± SEM of three independent experiments. Significant differences between each treatment and their respective controls (H_2_O_2_ non-treated cells): *P < 0.05, **P < 0.01, and ***P < 0.001. (C) Bar graph showing the proportion of DCF-fluorescent cells (indicating ROS production) at 15 minutes of exposure to different doses of H_2_O_2_ treatment. Bars represent the mean ± SEM of three independent experiments. Significant differences with respect to H_2_O_2_ non-treated cells: ***P < 0.001.

The response of hepatic cells to experimental oxidative stress was assessed by determining the viability of WRL68 cells at 24 h after treatment for 30 min with a single dose of H_2_O_2_ ranging from 0.25 to 5 mM. The MTT assay showed that cell viability decreased in a dose-dependent manner after oxidative treatment. Particularly, concentrations of less than 2.5 mM of H_2_O_2_ reduced cell viability by 30–40%, compared to the control (Fig 2A; 29.4 ± 2.7%, 32.8 ± 2.8% and 37.7 ± 1.1% reduction for 0.25, 0.5 and 1 mM, respectively). However, treatments at a concentration exceeding 2.5 mM of H_2_O_2_ provoked a decrease in cell survival of over 60% (Fig 2A; 63.2 ± 2.5% and 90.3 ± 0.2% reduction for 3.5 and 5 mM, respectively; 43.4 ± 4.4% reduction for 2.5 mM). To verify the generation of ROS in hepatic cells during H_2_O_2_ treatment, we analysed the conversion of H_2_DCFDA to DCF (see Methods). Time-course analysis of this oxidative treatment showed that the largest amounts of cells showing intracellular ROS were detected as early as 15 min after incubation with 3.5 mM of H_2_O_2_ and that these proportions of labelled cells were maintained until 30 min of H_2_O_2_ incubation, when the oxidative treatment concluded (Fig 2B). The number of cells with intracellular ROS dramatically decreased just a few minutes after the oxidative treatment. It is of note that the significant increase in cells with intracellular ROS was seen only at concentrations of H_2_O_2_ equal to or higher than 2.5 mM (Fig 2C; 84.6 ± 7.6% and 96.8 ± 4.7% increase for 2.5 and 3.5 mM, respectively). Therefore, the increased production of ROS observed after treatment with H_2_O_2_ (concentration ≥ 2.5 mM) correlates with a strong decrease in cell viability within 24 h.

### ROS induced by oxidative treatment activates PARPs

**Fig 3:**
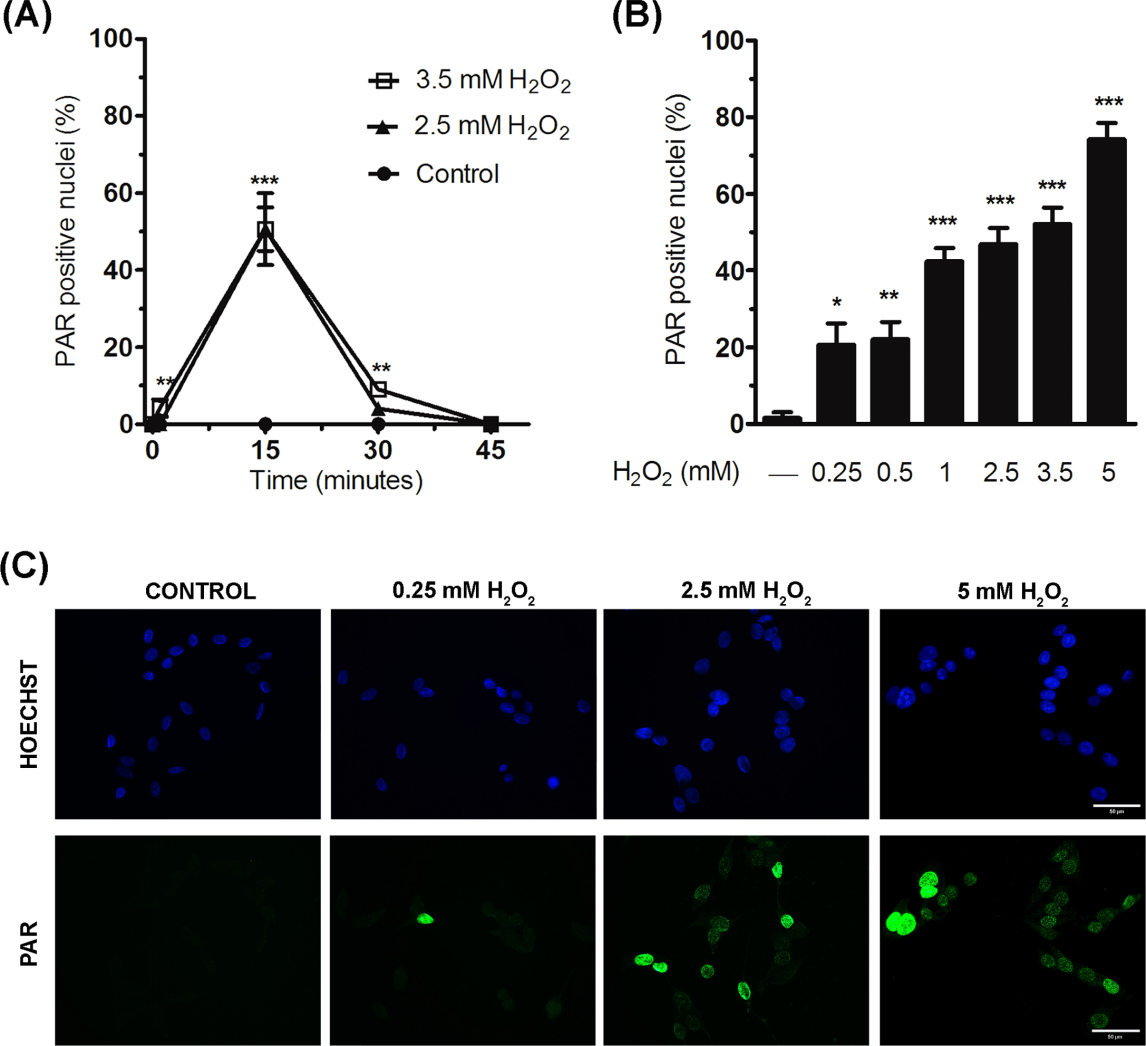
PARPs activation in response to oxidative treatment. (A) PARPs activity was measured by detecting PAR production by means of immunofluorescence. Cells were treated with H_2_O_2_ and PAR production was detected at 0, 15 and 30 min of exposure, and 15 minutes after 30 min of exposure to H_2_O_2_ (time point of 45 min in the graph). The linear graph shows the percentage of PAR positive nuclei (at least 200 cells were counted for each point and condition) at different time points; the highest level of PARPs activation was at 15 min of H_2_O_2_ treatment. Mean ± SEM of three independent experiments. Significant differences between each H_2_O_2_ treatment and their respective controls: **P < 0.01 and ***P < 0.001. (B) Bar graph revealing the effect of different concentrations of H_2_O_2_ on the percentage of PAR positive nuclei detected at 15 min of H_2_O_2_ exposure; at least 200 cells per condition were counted. Bars represent the mean ± SEM of three independent experiments. Significant differences with respect to H_2_O_2_ non-treated cells: *P < 0.05, **P < 0.01, and ***P < 0.001. (C) Representative immunofluorescence images showing the increase in PAR polymer (green) when WRL68 cells were treated with different doses of H_2_O_2_ for 15 min. Nuclei were stained with Hoechst (blue). Scale bar: 50 μm.

Oxidative stress can activate PARPs due to its ability to cause DNA breaks [35]. Therefore, we studied the levels of PARPs activation, measured by PAR polymer formation using immunofluorescence analysis, when ROS were present(Fig 3). We confirmed that most PAR polymer formation had taken place at 15 min from the beginning of H_2_O_2_ (Fig 3A), coinciding with the previously described early intracellular ROS production; hence, PARPs activation is an early event in these oxidative treatment. Particularly, PARPs activation increased as H_2_O_2_ concentration augmented (Fig 3B and 3C; 20.7 ± 5.5%, 22.0 ± 4.6%, 42.3 ± 3.5%, 45.3 ± 4.7%, 50.6 ± 4.0% and 72.6 ± 5.7% increase in PAR-positive cells for 0.25, 0.5, 1, 2.5, 3.5 and 5 mM H_2_O_2_ treatment compared to untreated control cells, respectively). So, treatment with H_2_O_2_ 3.5 mM increased early ROS production (96.8 ± 4.7% increase; Fig 2C), PARPs activation (50.6 ± 4.0% increase in PAR-positive cells; Fig 3) and strongly reduced cell viability at 24 h after the treatment (63.2 ± 2.5% reduction; Fig 2A).

### PARPs inhibitors increase cell viability and decrease the rate of cell death after oxidative treatment

We show above that cell viability strongly decreases with ROS induction (Fig 2) and PARPs activation (Fig 3) after 3.5 mM of treatment with H_2_O_2_. Therefore, we will use this concentration of H_2_O_2_ to study the role of PARPs activation on the hepatocytes suffering an oxidative insult.

**Fig 4:**
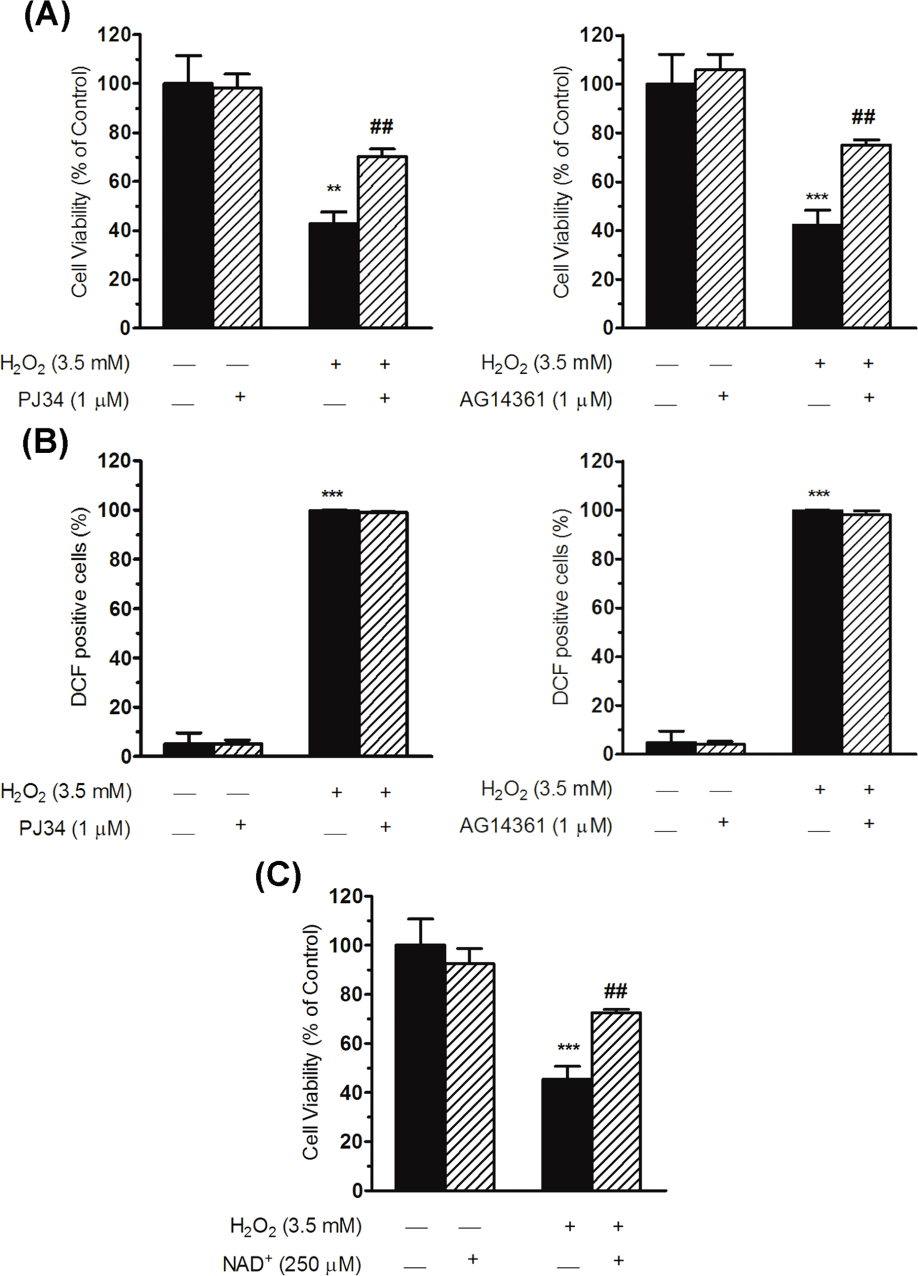
Effect of PARPs inhibitors or of NAD^+^ supplementation on hepatic cells after H_2_O_2_ treatment. (A) The viability of WRL68 cells (assessed with the MTT assay) was analysed after 16 h of pre-incubation, exposure to H_2_O_2_ for 30 min and 24 h of post-incubation, always in the presence of PARPs inhibitors. Cell viability was expressed as the percentage of viable cells with respect to those determined in the control (non-treated cells), which was considered to be 100%. Results from the PJ34 and AG14361 inhibitors are shown on bar graphs (left for PJ34, showing the mean ± SEM of five independent experiments; right for AG14361, showing the mean ± SEM of three independent experiments). Significant differences: **P < 0.01 and ***P < 0.001 with respect to the control (non-treated cells) and ^##^*P* < 0.01 with respect to 3.5 mM H_2_O_2_. (B) Bar graphs showing the percentage of cells containing ROS. The presence of ROS was measured by staining cells with H_2_DCFDA. WRL68 cells pre-incubated for 16 h with PARPs inhibitors were treated with H_2_O_2_ for 15 min and the proportion of cells positively marked with DCF fluorescence was recorded by flow cytometry. Bar graphs show the mean ± SEM of three independent experiments. Significant differences: ***P < 0.001 with respect to the control (non-treated cells). (C) Percentage of viable WRL68 cells treated with H_2_O_2_ for 30 min and 24 h of post-incubation, always in the presence of 250 μM of NAD^+^. Cell viability was determined by the MTT method and expressed as the percentage of viable cells with respect to those determined in the controls (non-treated cells), which was considered to be 100%. Bars represent the mean ± SEM of four independent experiments. Significant differences: ***P < 0.001 with respect to the control (non-treated cells) and ^##^*P* < 0.01 with respect to 3.5 mM H_2_O_2_.

In first place we determined whether PARPs inhibitors would abrogate the decrease in cell viability (Fig 4A). To do so, we used AG14361 (a potent and selective inhibitor of PARP-1 activity), which is known to inhibit over 90% of PARP-1 activity in human cells [36], and PJ34 (an inhibitor of PARP-1 and PARP-2 activity) at 1 μM concentration; higher concentrations were found to be cytotoxic in this cell line (S1 Fig). Both PARPs inhibitors produced a significant increase in the rate of cell survival after H_2_O_2_ treatment, with 27.3 ± 6.1% and 32.7 ± 5.5% increase in cell viability for PJ34 and AG14361 respectively (Fig 4A). Immunofluorescence techniques showed that PAR polymer formation was strongly diminished in hepatic cells treated with H_2_O_2_ and PARPs inhibitors (S2 Fig).

We speculated that PARPs inhibitors might increase cell survival rates due to the direct neutralisation of ROS or, alternatively, by preventing the depletion of NAD^+^ and hence the reduction in ATP, as a result of PARPs activation. To decide this question, we first examined whether ROS were formed in the presence of PARPs inhibitors. We found that ROS continued to be produced at normal levels despite the presence of the inhibitors of PARPs activity (Fig 4B); therefore both PJ34 and AG14361 have no scavenger capacity *per se* and, hence, their effect in recovering cell viability after oxidative damage is derived exclusively from the inhibition of PARPs activity. As a second approach, we considered whether NAD^+^ supplementation prevented a decrease in cell viability after the oxidative damage. To determine this point, hepatic cells were exposed to 3.5 mM H_2_O_2_ for 30 min, together with NAD^+^ incubation (0.25 mM), followed by 24 h of NAD^+^ post-incubation. NAD^+^ supplementation was found to significantly increase the cell survival rate of hepatic cells exposed to H_2_O_2_ treatment (27.2 ± 4.8% increase; Fig 4C), to a degree similar to that presented by the PARPs inhibitors.

**Fig 5:**
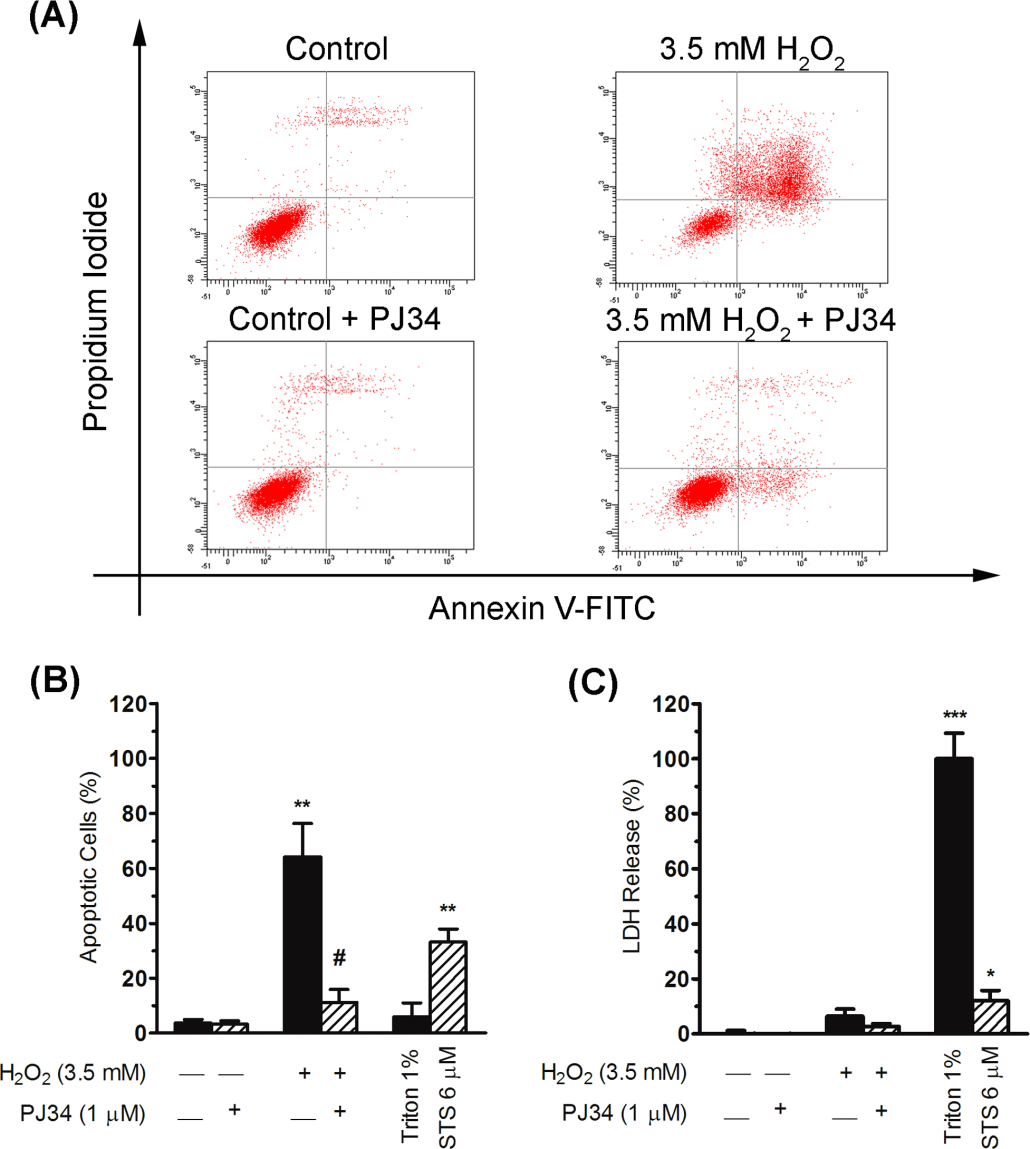
Cell death elicited by H_2_O_2_ treatment in the presence or absence of PARPs inhibitor PJ34. (A) Cell death was analysed by flow cytometry using Annexin-V-FITC and propidium iodide at 24 h after H_2_O_2_ exposure, in the presence or absence of PJ34 inhibitor. Dot plots from a representative flow cytometry experiment are shown. Cells in the respective quadrants are shown in red: lower left quadrants represent live cells; lower right, early apoptotic; upper left, necrotic cells; upper right, late apoptotic. (B) Percentage of apoptotic cells (lower and upper right quadrants in the dot plots) measured by detection of Annexin V-positive cells in the experimental conditions described above. Additionally, 6 μMstaurosporine (STS) treatment (for 24 h) was included as a positive control of apoptosis. Bar graph represents the mean ± SEM of three independent experiments. Significant differences: **P < 0.01 with respect to the control (non-treated cells) and ^#^*P* < 0.05 withrespect to 3.5 mM H_2_O_2_. (C) Lactate dehydrogenase (LDH) release was measured 24 h after H_2_O_2_ treatment in order to analysenecrosis in the presence or absence of PJ34 inhibitor. Additionally, 1% Triton treatment (for 10 min) was included as a positive control of necrosis. Bars show the mean ± SEM of three independent experiments. Significant differences: *P < 0.05 and ***P < 0.001 with respect to the control (non-treated cells).

The decrease in cell viability of hepatic cells exposed to H_2_O_2_ treatment was mainly correlated with a significant increase in Annexin V-positive cells (a marker of apoptosis; 60.5 ± 10.9% increase; Fig 5A and 5B). This apoptotic identity was corroborated by using staurosporine, a well-known apoptosis inducer [31], which markedly increased the number of Annexin V-positive cells (29.6 ± 4.7% increase; Fig 5B). In contrast, there was no such increase in LDH release, considered to indicate necrosis, after H_2_O_2_ treatment (6.4 ± 2.5% increase; Fig 5C). This suggests that a cell death mechanism similar to apoptosis (identify by the phosphatidylserine exposure detected with Annexin V and the low LDH release) may have occurred in hepatic cells when oxidative stress was induced. Interestingly, the PJ34 inhibitor significantly suppressed the increase in Annexin V-positive cells induced by H_2_O_2_ (52.9 ± 8.8% reduction; Fig 5A and 5B) but did not affect the LDH release after the oxidative insult (Fig 5C).

Taken together, these results show that a critical role is played by PARPs activation and NAD^+^ depletion in decreasing the survival rates of hepatic cells subjected to oxidative stress.

### PARPs inhibitors prevent the early alterations in mitochondrial form induced by ROS

**Fig 6:**
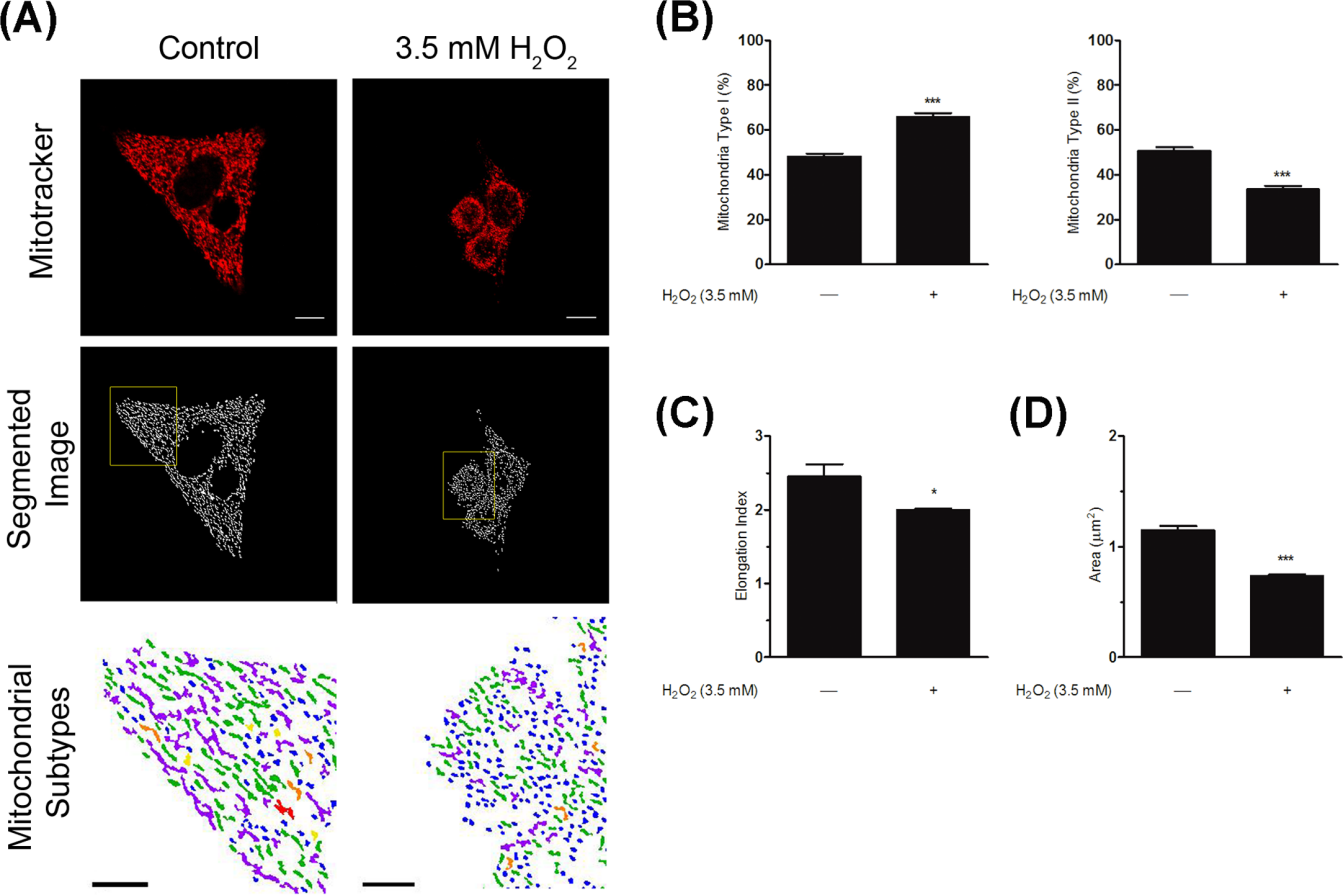
Changes in mitochondrial morphology after H_2_O_2_ treatment. (A) Confocal microscopy images of hepatic cells stained with Mitotracker (upper panel). Microscopy images show hepatic cells treated with H_2_O_2_ for 30 min and then post-incubated for 2 h. Mitotracker images were filtered and thresholded to obtain segmented images (middle panel) using MicroP software. The imagesegmentation method extracts each individual mitochondrial object from Mitotracker images to identify mitochondrial subtypes (lower panel; higher magnification of the boxed area in Segmented Image). MicroP software classifies morphological features as different colours according to their shape and size: blue, small globules; yellow, swollen globules; green, linear tubules; orange, twisted tubules; purple, branched tubules; and red, looped tubules. In this subtyping, fragmented mitochondria are represented by small globules (shown in blue). The composition of mitochondrial subtypes in the H_2_O_2_ treated cells was clearly modified with respect to the control cells. Scale bar in Mitotracker images: control, 10 μm; 3.5 mM H_2_O_2_, 18 μm. Scale bar in mitochondrial subtypes: control, 5 μm; 3.5 mM H_2_O_2_, 8 μm. (B) Bar graphs showing the percentage of type I (small globular; left graph) and type II (tubular including linear, twisted, branched and looped forms; right graph) mitochondrial morphology quantified with MicroP software in non-treated and H_2_O_2_ treated cells. (C) Bar graph showing the value of the mitochondrial elongation index (relation between major and minor axis lengths) in non-treated and H_2_O_2_ treated cells. Mitochondrial axis lengths were quantified with MicroP software. (D) Bar graph showing mitochondrial area obtained with MicroP analysis in non-treated and H_2_O_2_ treated cells. At least 7,000 mitochondria were analysed with MicroP software in each condition and in each experiment in (B), (C) and (D). Bars show the mean ± SEM of three independent experiments. Significant differences with respect to non-treated cells: *P < 0.05 and ***P < 0.001.

Oxidative stress induced by ROS overproduction has been related with mitochondrial damage and cell death [37, 38]. In our study, the rapid onset of ROS generation following exogenous H_2_O_2_ treatment induced changes in hepatic mitochondrial morphologies that have been associated with altered mitochondrial function [39]. The mitochondria were stained with Mitotracker and classified into two major morphological types using MicroP software at 2 h and 4 h after the exposure to H_2_O_2_: type I, corresponding to mitochondria with small globular shapes, and type II, mitochondria with tubular shapes. The mitochondria in the control cells were mainly organised into networks of tubular subtype forms (type II morphology) throughout the cytoplasm, and 2 h after H_2_O_2_ treatment, the mitochondria mainly presented small granular shapes (corresponding to type I morphology) and were predominantly located in the perinuclear region (Fig 6A). Most of the mitochondria in non-treated cells were tubular in shape (type II; 50.6 ± 1.7%), and 48.1 ± 1.4% were classified as type I (Fig 6B). This distribution was altered by H_2_O_2_ treatment, after which 65.9 ± 1.7% of the mitochondria had a type I morphology, while type II mitochondria represented only 33.5 ± 1.6% of the total (Fig 6B). Hence, at 2 h post-incubation after a single short-duration exposure to 3.5 mM H_2_O_2_, the presence of mitochondria morphology type I within the cells had increased by 17.8 ± 2.7%, while the mitochondrial type II morphology had decreased correspondingly. The change in mitochondrial morphology was additionally characterised by determining the elongation index of the mitochondria (the ratio of the lengths of the major and minor axes) and the area occupied by the individual mitochondria. Both parameters significantly decreased after H_2_O_2_ treatment, 18.1 ± 3.3% for the elongation index and 36.0 ± 1.7% for the area (Fig 6C and 6D). Mitochondrial shape and size alterations were even more marked between non-treated and H_2_O_2_-treated cells at 4 h after the treatment (S3 Fig): at this time, 77.3 ± 2.9% of the mitochondria were type I in H_2_O_2_ treated cells, a difference of 25.5 ± 2.1% with respect to the non-treated cells (S3A Fig). Coincidently, the mitochondrial elongation index and the area occupied also decreased after 4 h post-incubation (reductions of 21.8 ± 3.8% and 40.6 ± 1.8%, respectively; S3B and S3C Fig).

Hence, our results show that a single short exposure to high concentration of H_2_O_2_ induces alterations in the morphology and size of mitochondria in hepatic cells. These mitochondrial modifications are apparently related to mitochondrial fission and fragmentation processes, which have been extensively related with mitochondrial dysfunction and apoptosis. The alterations occurred relatively rapidly, as they were apparent only 2 h after H_2_O_2_ treatment, demonstrating that mitochondrial fragmentation is an early event in hepatic cells subjected to oxidative stress.

**Fig 7:**
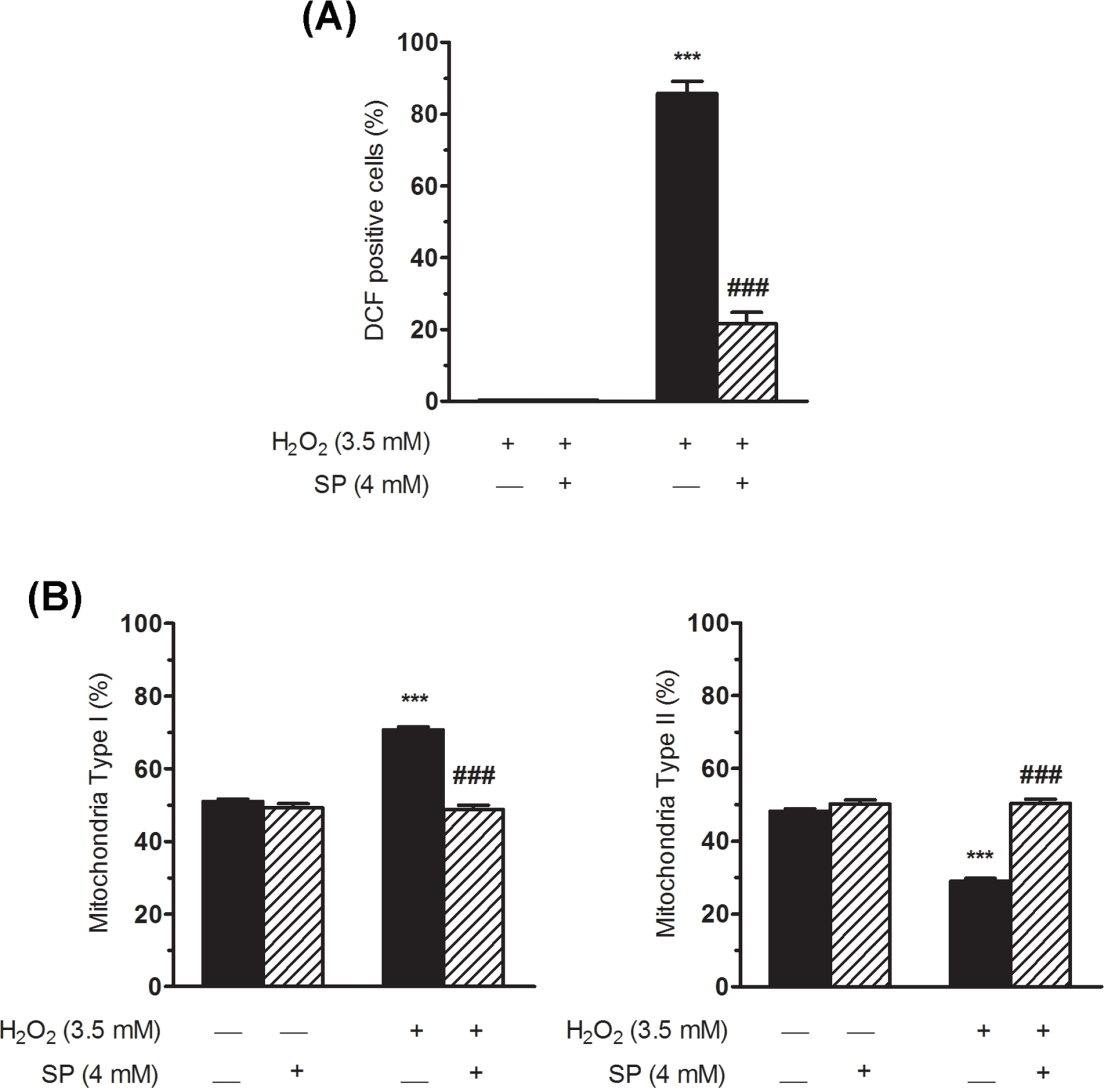
ROS scavenging counteracts mitochondrial fragmentation. (A) Percentage of DCF positive cells, evaluated by flow cytometry, at 15 minutes of exposure to 3.5 mM H_2_O_2_ in the presence or absence of 4 mM of sodium pyruvate (SP). (B) Bar graphs showing the percentage of type I (small globular; left graph) and type II (tubular including linear, twisted, branched and looped forms; right graph) mitochondrial morphology in each experimental condition. WRL68 cells were treated with H_2_O_2_ for 30 min and then post-incubated for 4 h prior to quantifying mitochondrial morphology with MicroP software. Sodium pyruvate (SP) was used in order to neutralise ROS production. At least 7000 mitochondria were analysed in each condition and in each experiment. Bars show the mean ± SEM of three independent experiments. Significant differences: ***P < 0.001 *versus* non-treated cells; ^###^*P* < 0.001 *versus* 3.5 mM H_2_O_2_.

To establish whether ROS production was involved in this mitochondrial fragmentation, sodium pyruvate was added to the culture medium to neutralise the ROS increase after H_2_O_2_ treatment; various studies have corroborated the cytoprotective properties of sodium pyruvate against oxidative stress through the direct scavenging of ROS [40–42]. The neutralisation of ROS by sodium pyruvate was confirmed by DCF detection (Fig 7A). Hepatic cells treated with H_2_O_2_ in the presence of this scavenger showed percentages of mitochondrial types I and II similar to those found in untreated cells (Fig 7B). Therefore, the mitochondrial fragmentation observed was related to increased levels of ROS within the cells.

**Fig 8:**
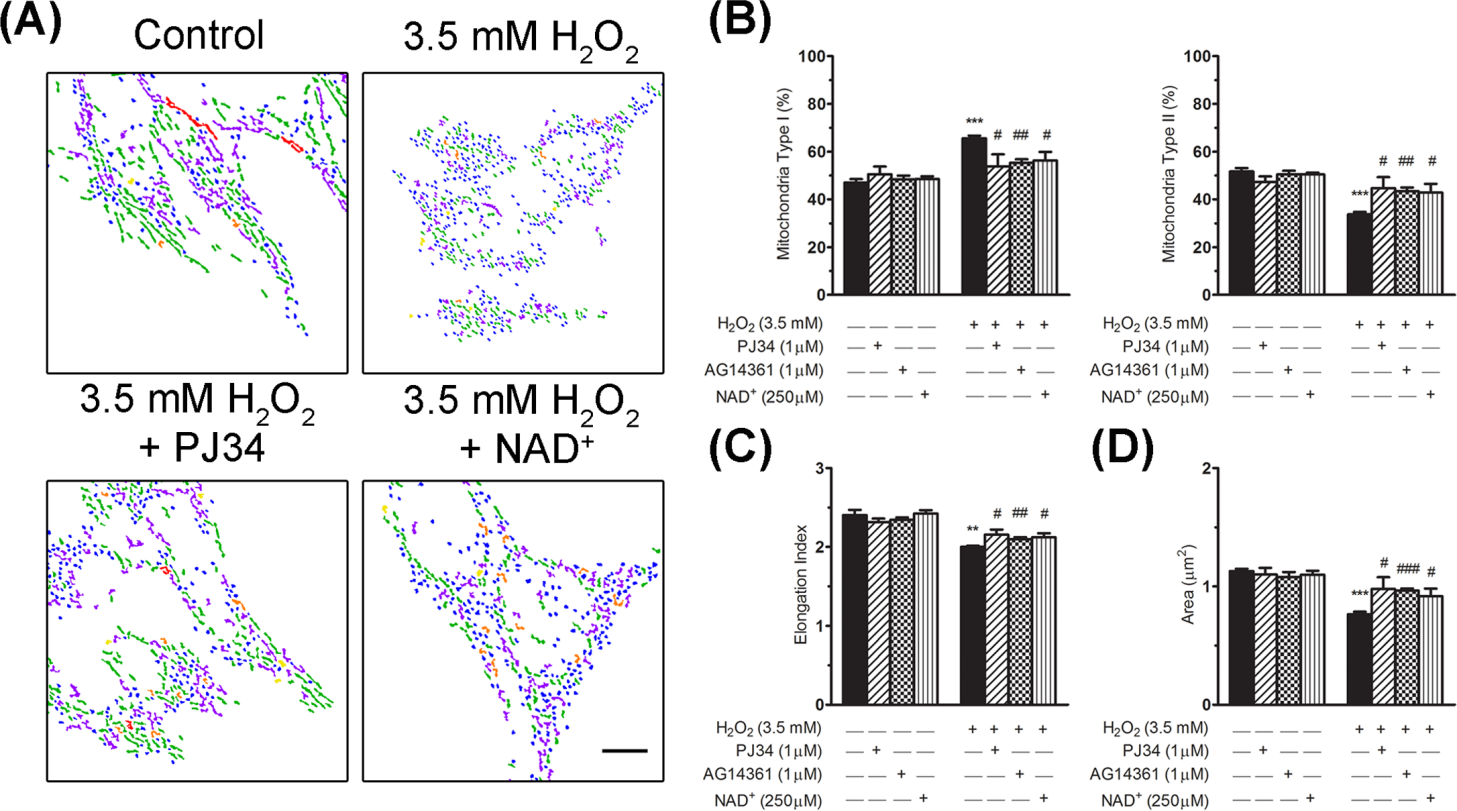
PARPs inhibition or NAD^+^ supplementation restores mitochondrial morphology after H_2_O_2_ treatment. (A) Composition of morphological subtypes of mitochondria (as described above, in the legend to Fig 6) at 2 h after exposure to 3.5 mM of H_2_O_2_, either in the presence or absence of PARPs inhibitors, and with or without NAD^+^ supplementation. Scale bar: 10 μm. (B) Bar graphs showing the percentage of type I (small globular; left graph) and type II (tubular including linear, twisted, branched and looped forms; right graph) mitochondrial morphology in each experimental condition. (C) Bar graph showing the value of the mitochondrial elongation index (relation between major and minor axis lengths) in each experimental condition. (D) Bar graph corresponding to the analysis of mitochondria area in each experimental condition. At least 7,000 mitochondria were analysed in each condition and in each experiment in (B), (C) and (D). Bars represent the mean ± SEM of four independent experiments. Significant differences: **P < 0.01 and ***P < 0.001 *versus* control (non-treated cells); ^#^*P* < 0.05, ^##^*P* < 0.01 and ^###^*P* < 0.001 *versus*3.5 mM H_2_O_2_.

In the next stage of this study, we analysed the effect of PARPs inhibitors on the morphological alterations detected in mitochondria after the exposure to H_2_O_2_. The quantification of mitochondrial shape subtypes (Fig 8A) showed that both the PJ34 and the AG14361 PARPs inhibitors significantly reduced the increase in mitochondrial type I morphology (12.4 ± 5.2% and 9.9 ± 1.5% reduction, respectively) caused by oxidative stress at 2 h post-incubation (Fig 8B). Our analysis also showed that cells treated with H_2_O_2_ and PARPs inhibitors displayed a significantly higher percentage of type II mitochondria morphology (11.6 ± 4.7% and 9.4 ± 1.5% increase, for PJ34 and AG14361, respectively) in comparison with cells treated only with H_2_O_2_ (Fig 8B). Moreover, the elongation index and the mitochondrial area were also significantly increased in oxidative-stressed cells incubated with PARPs inhibitors (Fig 8C and 8D). NAD^+^ supplementation caused effects on mitochondrial size and morphology that were similar to those of the PARPs inhibitors after the H_2_O_2_ insult (Fig 8B–8D), suggesting that the NAD^+^ depletion caused by PARPs activation plays a critical role in alterations in mitochondrial morphology and size. The effect of the PARPs inhibitors on mitochondrial shape and size was most evident at 4 h post-incubation after the oxidative insult (S4 Fig).

**Fig 9:**
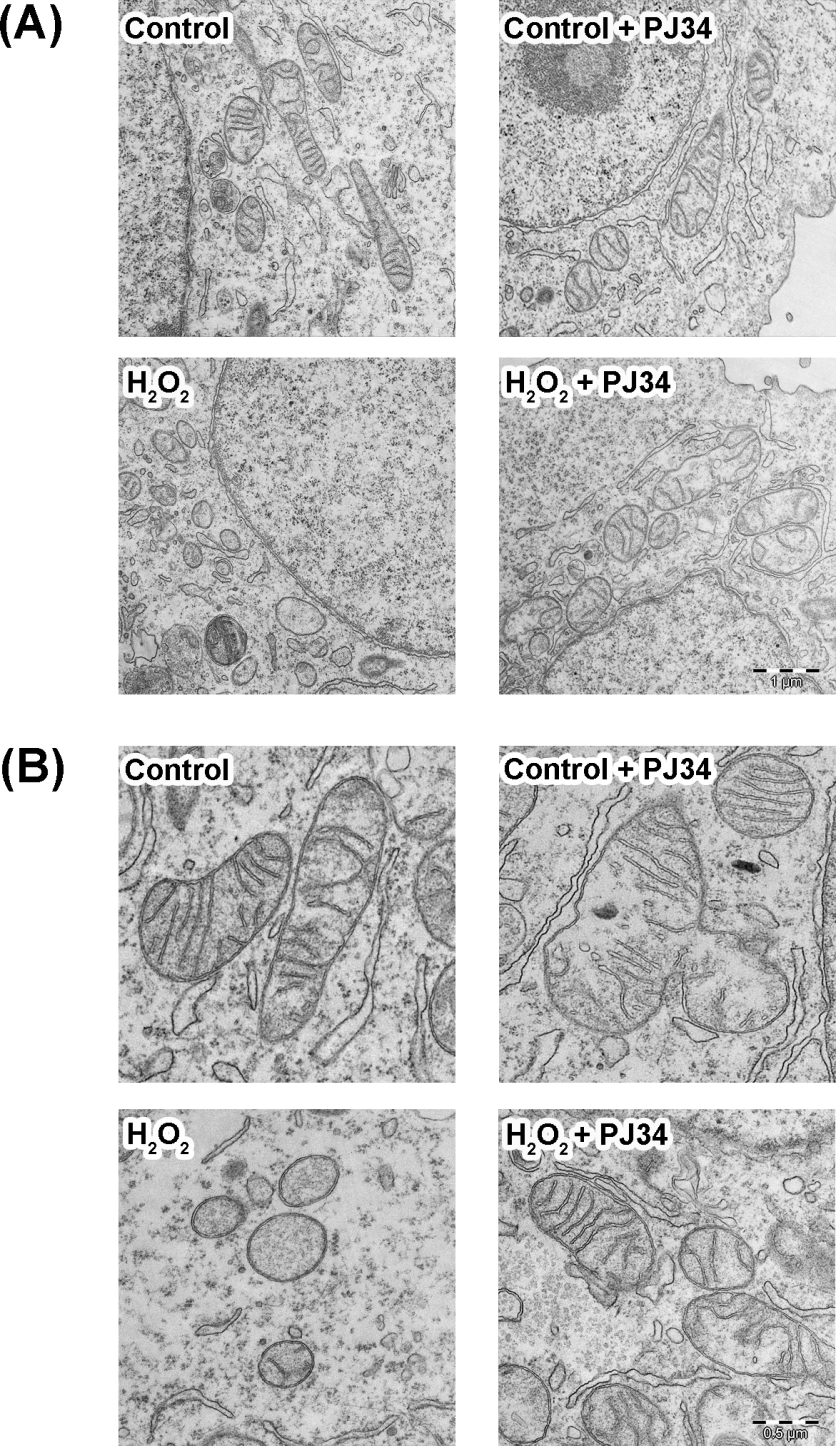
Analysis of mitochondrial ultrastructure. Transmission electron microscope photomicrographs corresponding to thin sections of WRL68 cells at 4000x (A) and 8000x magnification (B). Cells were pre-incubated, treated with H_2_O_2_ for 30 min and then post-incubated for 2 h (in the presence or absence of PJ34 inhibitor) before being analysed by electron microscopy. The H_2_O_2_ treated cells, in the absence of PJ34 inhibitor (lower left photomicrographs), show mitochondria with small granular morphology and scarce transversal cristae, while the presence of the inhibitor in oxidative treatment (lower right photomicrographs) restores the tubular-like morphology and transversal cristae distribution. Scale bar: 1 μm in (A) and 0.5 μm in (B).

To determine whether the alterations in mitochondrial morphology and size previously evidenced with Mitotracker staining were related to ultrastructural alterations in the mitochondria, both treated and untreated cells were processed for TEM (Fig 9). In hepatic cells non-treated with H_2_O_2_, irrespective of whether they had been incubated with PARPs inhibitors, the mitochondria mainly showed a tubular-like and rounded morphology with clearly defined transversal cristae (Fig 9A and 9B; top panels). However, after 2 h of treatment with 3.5 mM H_2_O_2_, the hepatic cells showed many small mitochondria globules and fewer mitochondrial cristae (Fig 9A and 9B; bottom left panel); these ultrastructural morphologies have been related with mitochondrial fission and fragmentation. Interestingly, hepatic cells subjected to H_2_O_2_ treatment in the presence of PARPs inhibitors (Fig 9A and 9B; bottom right panel) conserved similar mitochondria morphologies to those of the H_2_O_2_ untreated cells. Therefore, the results about the recovery of mitochondrial alterations in presence of PARPs inhibitors obtained with Mitotracker staining and confocal microscopy were corroborated at the ultrastructural level.

In summary, the above results clearly suggest that PARPs inhibitors prevent the early changes in mitochondrial and ultrastructural morphologies associated with oxidative stress induced by a single short exposure to H_2_O_2_.

### AG14361 inhibitor counteracts the decrease in ATP induced by ROS

**Fig 10:**
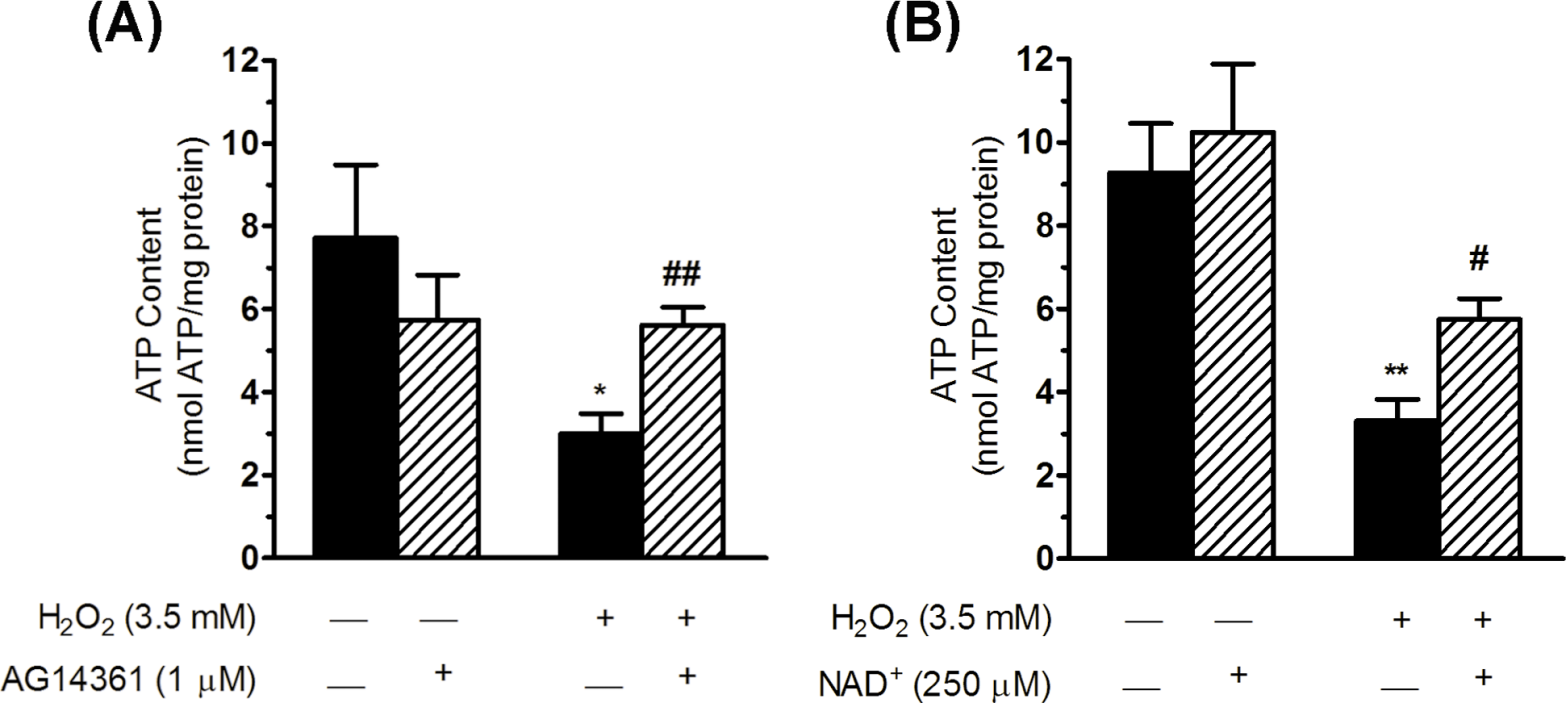
PARPs inhibition or NAD^+^ supplementation increases the ATP cell content after H_2_O_2_ treatment. (A) The bar graph shows the ATP content (in nmol per mg of total protein) obtained in soluble extracts from WRL68 cells in each experimental condition. Hepatic cells were pre-incubated, treated with H_2_O_2_ for 30 min and post-incubated for 2 h (always in the presence of AG14361 inhibitor) before being processed to obtain the cell extracts. (B) NAD^+^ was supplemented during oxidative treatment (30 min) and during the post-incubation time (2h). Then, cell extracts were obtained and processed to measure ATP content (in nmol per mg of total protein). Bars represent the mean ± SEM of four (A) and three (B) independent experiments. Significant differences: *P < 0.05 and **P < 0.01 *versus* control (non-treated cells); ^#^*P* < 0.05 and ^##^*P* < 0.01 *versus* 3.5 mM H_2_O_2_.

Mitochondrial fragmentation and the apparent reduction in mitochondrial cristae observed in H_2_O_2_ treated cells have been closely related to defects in energy production and, hence, to mitochondrial dysfunction. In order to determine the impairment of mitochondrial function, we next analysed cellular ATP content, using an ATP bioluminescence assay performed on intact cells treated for 30 min with H_2_O_2_ either in the absence or presence of PARPs inhibitors (see Methods). A significant decrease in ATP content was observed at 2 h after the H_2_O_2_ treatment (a 4.7 ± 1.4 nmol/mg reduction compared with non-treated cells; Fig 10A), which was significantly neutralised with the AG14361 inhibitor (2.6 ± 0.2 nmol/mg increase in ATP content compared with H_2_O_2_ treatment; Fig 10A). Concordantly, NAD^+^ supplementation in oxidative treatment also caused an increase in ATP content similar to those observed for the AG14361 inhibitor (2.4 ± 0.6 nmol/mg increase compared with H_2_O_2_ treatment; Fig 10B), suggesting that NAD^+^ depletion caused by PARPs activation contributes to mitochondrial energetic dysfunction.

## Discussion

The present study investigates the effect of the PARPs inhibitors PJ34 (which inhibits PARP-1 and PARP-2) and AG14361 (which specifically inhibits PARP-1) on cell survival and death after oxidative insult. The oxidative insult of WRL68 cells, used as an *in vitro* hepatic model [43], consisted in a short treatment with H_2_O_2_. As PARP-1 and PARP-2, whose overactivation produces the depletion of cellular NAD^+^, are involved in the sensing and repair of DNA, their effect on cell death and survival was analysed by inhibiting their activity. The survival of WRL68 cells following H_2_O_2_ treatment increased when the PARPs were inhibited. Moreover, since both inhibitors recovered similar amounts of cell viability, we concluded that this effect is essentially due to the inhibition of PARP-1 activity. In addition, we demonstrate that the increased cell survival could be due to preventing the NAD^+^ depletion produced after the PARPs activation induced by oxidative stress. Similar results have been obtained in chick embryo liver and other types of cells characterised by PARP-1 overactivation and supplemented with NAD^+^ [44–46]. As the depletion of NAD^+^ has been related to a loss of ability to regenerate ATP within the cells, its restoration or the prevention of its depletion by inhibiting PARPs enzymes could avoid energy failure in hepatic cells.

We used millimolar concentrations of H_2_O_2_ to induce oxidative stress in our *in vitro* model, which are higher than physiological and most pathological concentrations. We made this choice after finding that WRL68 hepatic cells were resistant to cell death induced by micromolar H_2_O_2_ concentrations. This resistance may be related to the elaborate detoxification and antioxidant systems of hepatic cells, likely developed to maintain cellular homeostasis in the liver[47]. In contrast, other cell types are more sensitive to H_2_O_2_ treatment, and concentrations above 50 μM have been described as cytotoxic to a wide range of animal cells in culture[48]. Hence, the H_2_O_2_ dosage must be adapted to each cell type. In summary, the concentration of H_2_O_2_ selected (3.5 mM concentration for 30 min) resulted in a strong reduction in cell viability (Fig 2A), ROS accumulation in all cells (Fig 2C), an increase in PARPs activation (Fig 3) and mitochondrial alterations (Fig 6). These mitochondrial alterations were similar to those documented in various liver diseases[14, 15, 49, 50]. The present study presents a possible experimental model for the study of these changes and the relationships among ROS production, PARPs activation and mitochondrial alteration. Further research is warranted on the potential role of PARPs in the suppression of oxidative stress-associated cell death in hepatic diseases that develop with early mitochondrial changes.

The inhibition of PARPs is of clinical interest. These inhibitors have been described as a powerful therapy for patients with hereditary breast or ovarian cancer containing mutations in the BRCA-1 and BRCA-2 genes that are essential for repairing DNA lesions by the homologous recombination pathway [51, 52]. The inhibition of PARPs in these genetic conditions enhances the formation of non-repaired double strand breaks, producing cytotoxic lesions that induce cell death in the cancer cells [53]. Additionally, PARPs inhibitors have been related to potential therapeutic effects in non-oncological diseases [54]; in anti-inflammatory therapy [22, 24, 55] they reduce the NAD^+^ depletion that triggers necrotic cell death and antagonise the transcription of pro-inflammatory genes promoted by PARPs [24, 25]. Our findings, from using WRL68 cells (as an *in vitro* model of hepatic cells), corroborate the therapeutic possibilities of PARPs inhibitors in disorders linked to oxidative stress as a means of reducing NAD^+^ and ATP depletion. Therefore, our results agree with that of other authors, that the pharmacological inhibition of PARP-1 is a promising therapy for hepatic cells suffering oxidative stress, as well as for inflammatory disorders and bioenergetic dysfunction [56–58].

The result of PARP-1 overactivation is cell death. Depletion of NAD^+^ and/or ATP after PARPs activation may originate necrotic cell death [23]. In addition, PAR is involved in the release of AIF from mitochondria to the nucleus that induces apoptosis [59]. In our study, 30 min of exposure to 3.5 mM H_2_O_2_ (referred to in the text as short-exposure time) caused death in WRL68 liver cells that was characterised as apoptosis, as the cells expressed biochemical markers of apoptosis, such as phosphatidylserine exposure on the outer leaflet of the plasma membrane; however, translocation of the AIF to the nucleus was not detected (data not shown). According to a study in which the same hepatic cell line was treated for 3 h with 0.3 mM H_2_O_2_, the PJ34 inhibitor increases Akt activation, which in turn inhibits the proapototic molecule BAD [60]. It should be noted that both the latter study and our own recorded an increase in the viability (evaluated by the MTT assay) of cells treated with a PARPs inhibitor, although we did not observe a relation between the activation of Akt and cell survival (data not shown). Finally, a third study [56], which used mouse primary hepatocytes treated with 3 mM H_2_O_2_ for 16 h (long exposure time), described an increase in necrosis that was reduced by PARPs inhibitors. Hence, it seems that the mechanisms implicated in H_2_O_2_ induced cell death in hepatic cells depend on the concentration and duration of the oxidative treatment.

Oxidative stress induced by exogenous H_2_O_2_ treatment leads to multiple cell damage, producing an increase in ROS and in its reaction with cellular components such as protein, lipids and nucleic acids. Hence, oxidative stress and ROS can have various biological consequences, which include non-survival or survival according to the extent of damage and the cellular context[61]. The resultant DNA oxidative damage can lead to mild activation or overactivation of PARPs. In the first instance, PARPs activation induced by moderate oxidative damage does not cause depletion of NAD^+^, and the cell death produced would not depend on PARPs activation. However, excessive oxidative stress can elicit extensive DNA damage associated with a strong NAD^+^ depletion generated by PARPs overactivation, and the ensuing cell death would be PARPs-dependent[22, 62, 63]. The present results are consistent with this proposition, given that lower doses of H_2_O_2_ (0.25 mM) were associated with low ROS generation (Fig 2C), moderate PARPs activation (Fig 3B) and no mitochondria alteration (data not shown), producing a lesser decrease in cell viability (Fig 2A), whereas higher H_2_O_2_ concentrations (3.5 mM; Fig 2A) markedly reduced the proportion of viable cells, generating elevated ROS production (Fig 2C), PARPs overactivation (Fig 3B) and mitochondrial dysfunction (Fig 6). These data suggest that the decreased cell viability at low H_2_O_2_concentrations is independent of PARPs activation, while the increased cell death at higher H_2_O_2_ concentrations results from PARPs overactivation. Therefore, PARPs are not involved in all of the cell death phenomena, as indicated by the fact that PARPs inhibitors did not rescue all cell death, and PARPs-independent cell death mechanisms would also play a role after oxidative damage.

Nevertheless, PARPs inhibitors conferred protection against cell death to a substantial percentage of cells, and research is warranted on its usefulness in therapies in which hepatic cells are exposed to high ROS concentrations.

A major point of our findings is the relationship observed between alterations in mitochondrial morphology and the energetic dysfunction detected after oxidative treatment. *In vivo* observations suggest that mitochondrial dysfunction is intimately linked to the bioenergetics status in metabolic diseases of hepatic cells [15]. Mitochondrial dysfunction is related to changes in the morphology of mitochondria, whichranges from long interconnected tubules to small globular forms, depending on the physiological condition [17], with the small globular forms being related to the process of mitochondrial fragmentation. Our extensive analysis of the mitochondrial alterations induced by oxidative stress and ROS in hepatic cells shows that mitochondrial fragmentation and loss of cristae are an early and progressive event after oxidative stress, and that these alterations are counteracted by PARPs inhibition.

Mitochondrial fission lowers respiratory activity, ATP cell content, mitochondrial dysfunction and cell death [16]. Previous *in vitro* studies revealed that mitochondrial fragmentation is enhanced by oxidative stress and ROS [64–68]. Interestingly, we show, too, that the mitochondrial fragmentation and energetic dysfunction (loss of ATP content) induced by ROS production after H_2_O_2_ treatment are inhibited by PARPs inhibitors in an *in vitro* model of hepatic cells. In short, ROS formation, PARPs activation, mitochondrial fragmentation and cell death appear to be causally related. A recent work using retinal cells reported a similar correlation [69].

PARPs activation apparently impairs mitochondrial function [70–74], but the mechanism underlying this effect has not been elucidated and several possibilities have been proposed. In the first place, as the NAD^+^ coenzyme is essential for glycolysis, the tricarboxylic acid cycle and mitochondrial respiration, its depletion after PARPs activation would result in an energetic catastrophe within the cell, and, hence, in mitochondrial dysfunction. Secondly, some authors have proposed that, in addition to nuclear activity, there exists a mitochondrial PARPs activity that could promote mitochondrial dysfunction by the poly-ADP-ribosylation of mitochondrial proteins [73, 74]. Third, we also hypothesise that mitochondrial impairment after PARPs activation may be related to alterations in the NAD^+^/NADH pool, as NAD^+^ enhances the biogenesis of mitochondrial respiratory chain complexes [75] in addition to playing a role in mitochondrial energy production [76]. NAD^+^ is reduced to NADH, and NADH, in turn, enhances the import and biogenesis of respiratory chain complexes; therefore, the reduction of NADH after NAD^+^ depletion would affect the mitochondria. Finally, PARPs activation-induced NAD^+^ depletion may interfere with NAD^+^-dependent deacetylase sirtuin-3 (SIRT3), which is a member of the sirtuin enzyme family located in the mitochondria. SIRT3 is highly expressed in the liver and is crucial for the maintenance of mitochondrial functions[77]. SIRT3 function in the mitochondria is essential for regulation of the activities of respiratory complexes, the antioxidant enzyme MnSOD and other enzymes involved in regenerating reduced cofactors in order to maintain a proper glutathione redox status[77]. SIRT3 therefore has a crucial role in energy production and ROS detoxification. SIRT3 probably competes with PARPs for NAD^+^, and PARPs overactivation has been found to correlate with sirtuin activity downregulation[78].Conversely, PARP-1 inhibition restores the activity of SIRT3 and various mitochondrial antioxidant enzymes[79]. Therefore, the mechanism by which PARPs inhibition could prevent mitochondrial dysfunction and fragmentation might be related to the participation of SIRT3 in maintaining mitochondrial functions. Further studies are necessary to establish the particular role played by PARPs activation in the fragmentation of mitochondria and in energetic dysfunction.

**Fig 11:**
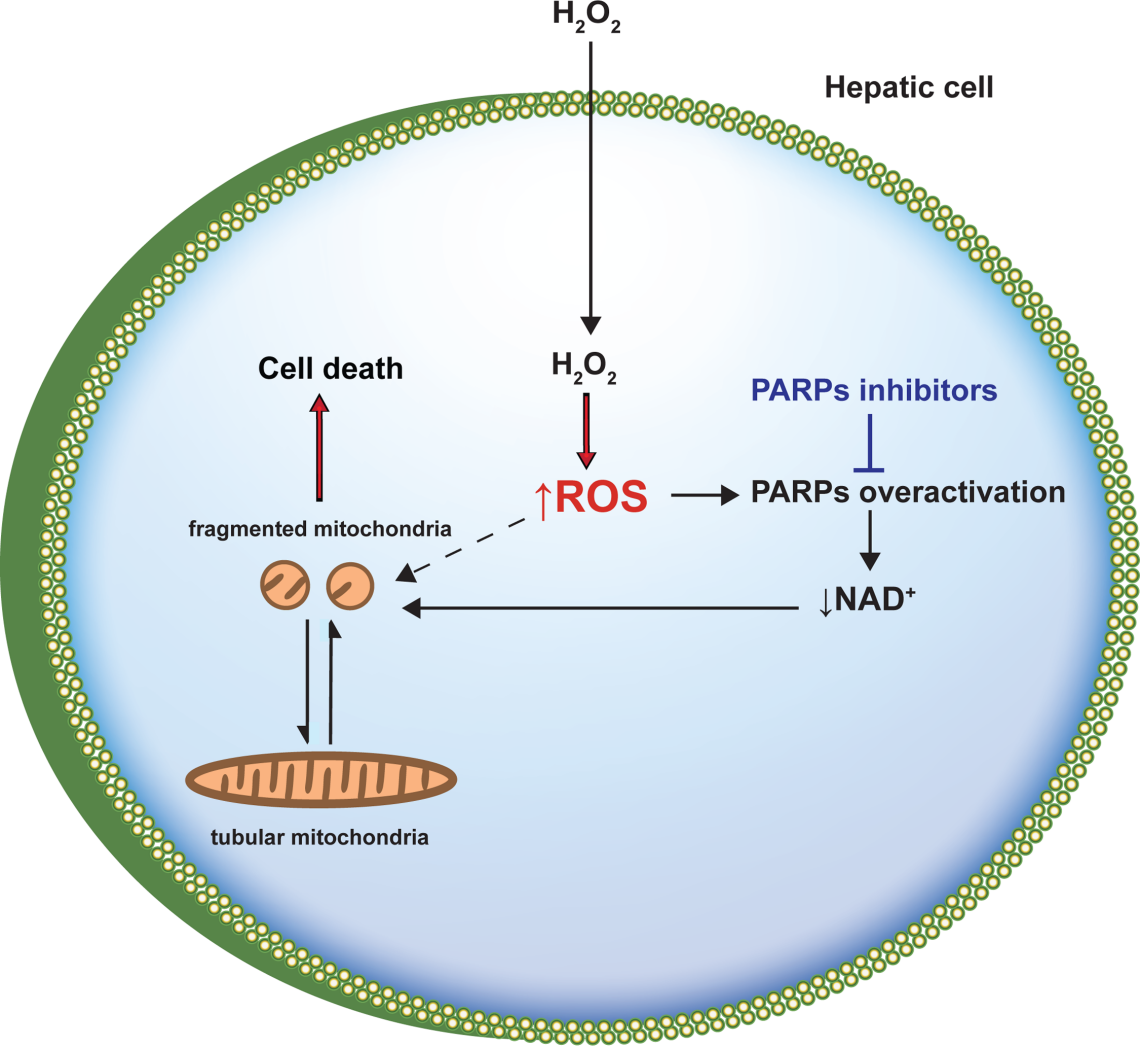
Scheme showing the proposed mechanism by which PARPs inhibitors prevent mitochondrial fragmentation and decrease cell death induced by ROS in hepatic cells. Reactive oxygen species (ROS) induced by oxidative treatment (H_2_O_2_) stimulated PARPs activation, generating NAD^+^ depletion and causing an alteration in the NAD^+^/NADH pool. This alteration produces changes in mitochondrial morphology, resulting in mitochondrial fragmentation and cell death as a consequence. The inhibition of PARPs activity by PARPs inhibitors avoid NAD^+^ depletion and therefore prevents mitochondrial fragmentation and cell death.

In conclusion, this study presents some novel and interesting data regarding the change in mitochondrial morphology after PARPs inhibition and its relationship with the decrease of hepatic cell death induced by ROS, which are summarized in Fig 11. We report that mitochondrial fragmentation is an early event in hepatic cells suffering oxidative stress causing cell death. PARPs inhibitors reduce this cell death, by protecting against mitochondrial fragmentation and NAD^+^ depletion, and restore mitochondrial function.

## Supporting information

S1 Fig Study of cytotoxic effects of PARPs inhibitors in WRL68 cells. Effects of PARPs inhibitors on the cell viability of WRL68 cells assessed by the MTT method. Cell viability was determined in WRL68 cells incubated with PJ34 or AG14361. To do so, hepatic cells were exposed for 40 h (16 h of pre-treatment and 24 h of post-incubation time) to different concentrations (0.1, 0.5, 1, 10 mM) of PARPs inhibitors as indicated. Cell viability was expressed as percentages of the control, which was considered to be 100%. Mean ± SEM of three independent experiments. Significant differences with respect to the control (non-treated cells): *P < 0.05.
(TIF)

S2 Fig PARPs inhibitors block cellular PAR polymer formation. Immunofluorescence detection of PAR polymer (green) in WRL68 cells treated with H_2_O_2_ for 15 min in the absence or presence of the PJ34 inhibitor. Immunofluorescence images show that PAR polymer formation is blocked when PARPs inhibitors were used in oxidative treatment. Nuclei were counterstained with Hoechst (blue). Representative images from three independent experiments. Scale bar: 50 μm.
(TIF)

S3 Fig Morphology of mitochondria 4 h after H_2_O_2_ treatment. (A) Percentage of type I (small globular; left graph) and type II (tubular including linear, twisted, branched and looped forms; right graph) of mitochondrial morphology. Cells were treated with H_2_O_2_ for 30 min and then post-incubated for 4 h prior to quantifying mitochondrial morphology with MicroP software. (B) Mitochondrial elongation index (relation between major and minor axis lengths). (C) Mitochondrial area. Bars represent the mean ± SEM of four independent experiments; at least 7,000 mitochondria were analysed in each condition and in each experiment. Significant differences with respect to non-treated cells: ***P < 0.001.
(TIF)

S4 Fig PARP inhibition restores mitochondrial morphology 4 h after H_2_O_2_ treatment. (A) Percentage of type I (small globular; left graph) and type II (tubular including linear, twisted, branched and looped forms; right graph) of mitochondrial morphology prior to quantifying mitochondrial morphology with MicroP software. WRL68 cells pre-incubated 16 h with AG14361 were treated with H_2_O_2_ for 30 min and then post-incubated for 4 h. (B) Elongation index of mitochondria. (C) Area of mitochondria. At least 7000 mitochondria were analysed with MicroP software in each condition and in each experiment. Bars represent the mean ± SEM of three independent experiments. Significant differences: **P < 0.01 with respect to the control (non-treated cells); ^#^*P* < 0.05 with respect to 3.5 mM H_2_O_2_.
(TIF)
