# Supplementary figures and images for: Poly(ADP-ribose)polymerases inhibitors prevent early mitochondrial fragmentation and hepatocyte cell death induced by H2O2
Source: PLoS One. 2017 Oct 26;12(10):e0187130. doi: 10.1371/journal.pone.0187130 (PMC5658148; doi:10.1371/journal.pone.0187130)

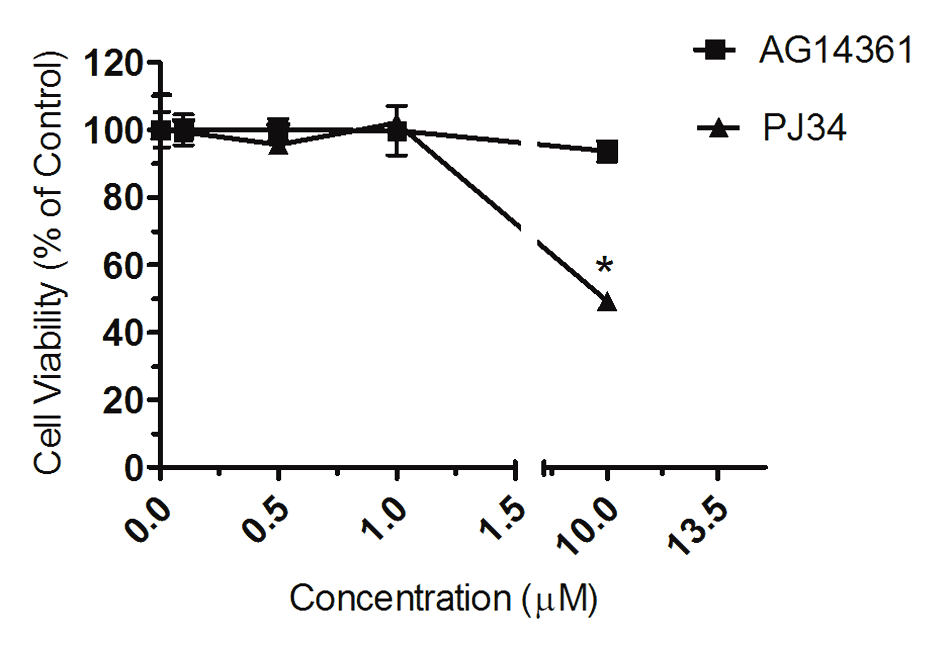

Supplement: S1 Fig — Effects of PARPs inhibitors on the cell viability of WRL68 cells assessed by the MTT method. Cell viability was determined in WRL68 cells incubated with PJ34 or AG14361. To do so, hepatic cells were exposed for 40 h (16 h of pre-treatment and 24 h of post-incubation time) to different concentrations (0.1, 0.5, 1, 10 mM) of PARPs inhibitors as indicated. Cell viability was expressed as percentages of the control, which was considered to be 100%. Mean ± SEM of three independent experiments. Significant differences with respect to the control (non-treated cells): *P < 0.05. (TIF) [file pone.0187130.s001.tif]

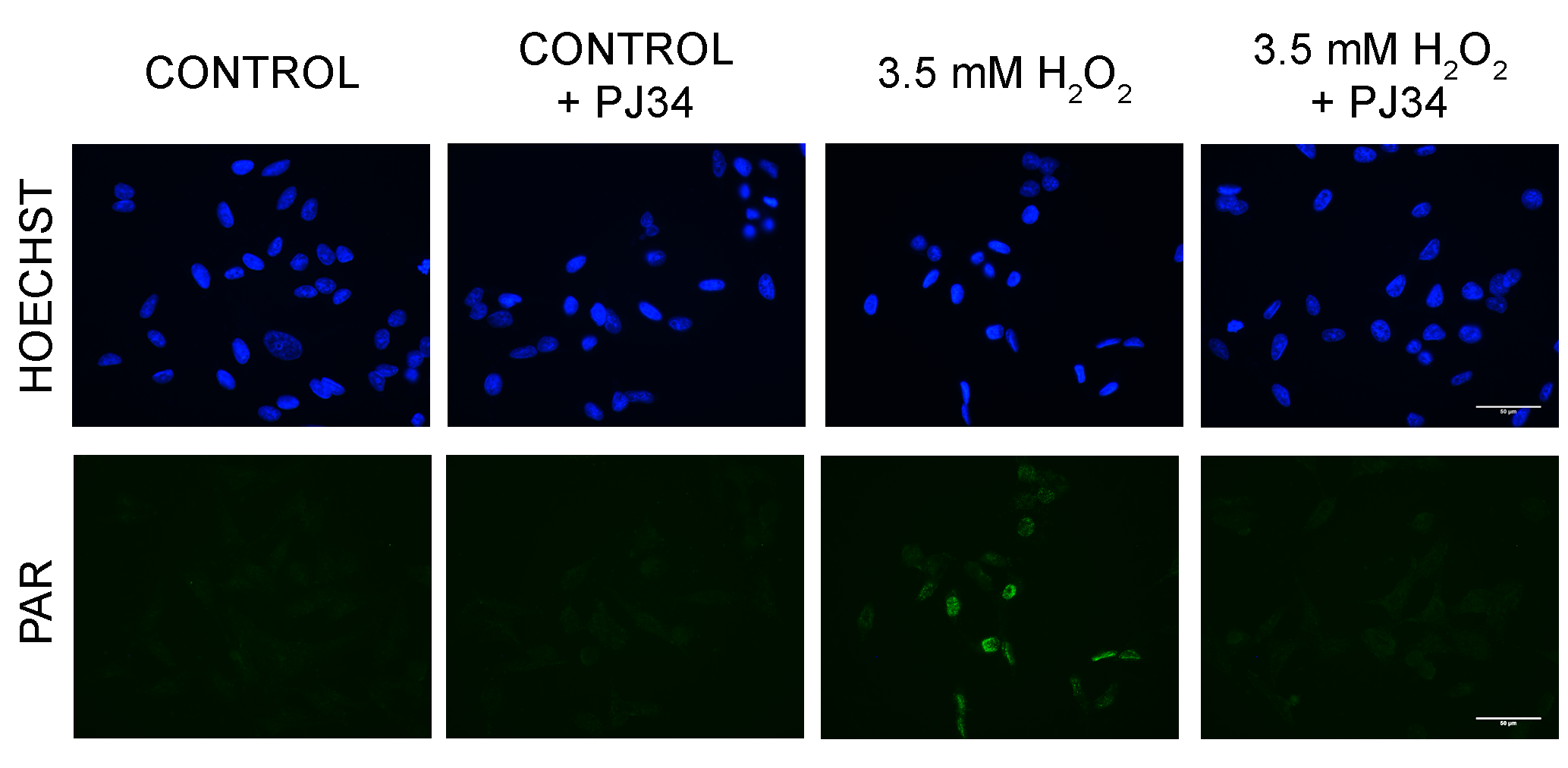

Supplement: S2 Fig — Immunofluorescence detection of PAR polymer (green) in WRL68 cells treated with H2O2 for 15 min in the absence or presence of the PJ34 inhibitor. Immunofluorescence images show that PAR polymer formation is blocked when PARPs inhibitors were used in oxidative treatment. Nuclei were counterstained with Hoechst (blue). Representative images from three independent experiments. Scale bar: 50 μm. (TIF) [file pone.0187130.s002.tif]

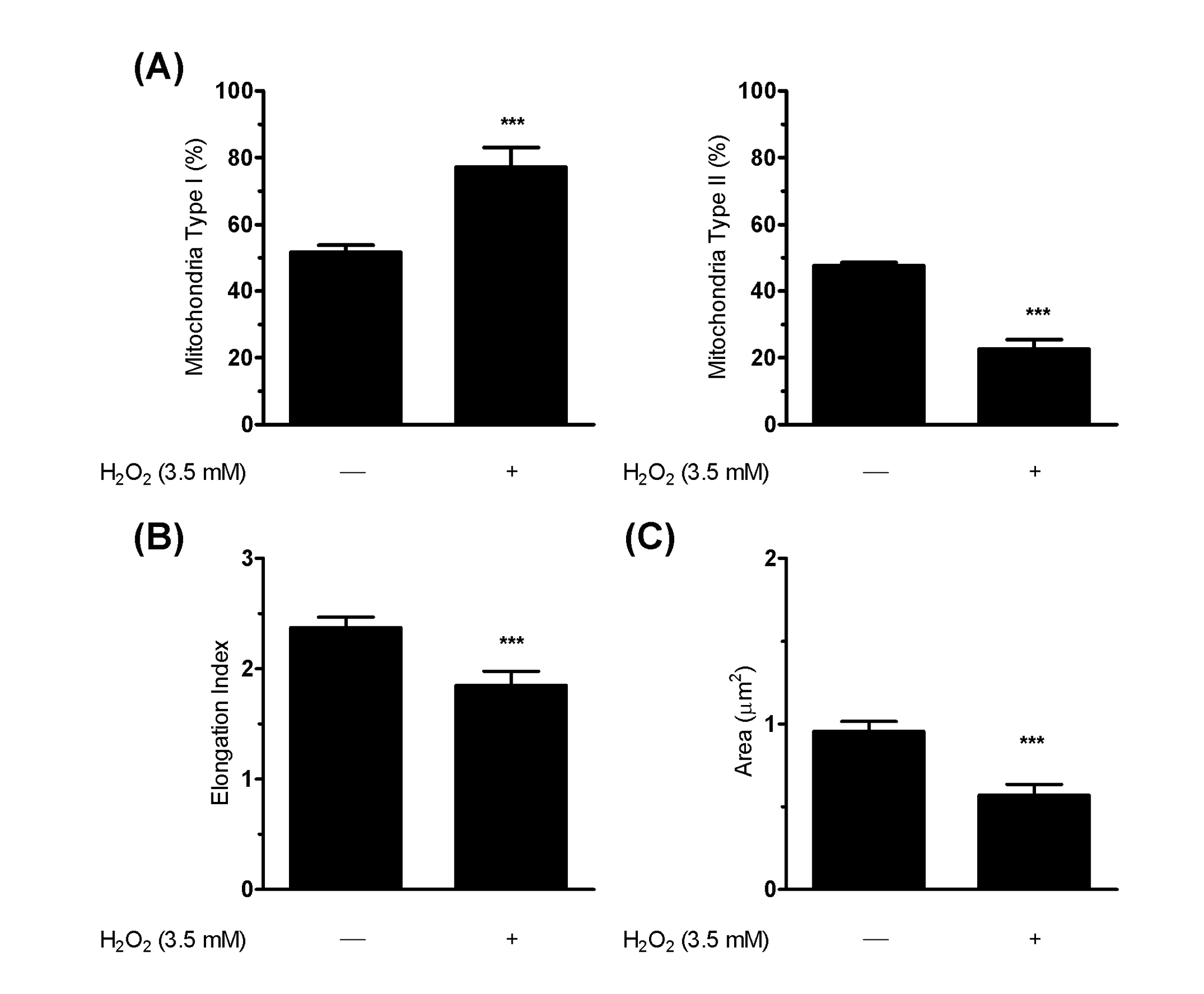

Supplement: S3 Fig — (A) Percentage of type I (small globular; left graph) and type II (tubular including linear, twisted, branched and looped forms; right graph) of mitochondrial morphology. Cells were treated with H2O2 for 30 min and then post-incubated for 4 h prior to quantifying mitochondrial morphology with MicroP software. (B) Mitochondrial elongation index (relation between major and minor axis lengths). (C) Mitochondrial area. Bars represent the mean ± SEM of four independent experiments; at least 7,000 mitochondria were analysed in each condition and in each experiment. Significant differences with respect to non-treated cells: ***P < 0.001. (TIF) [file pone.0187130.s003.tif]

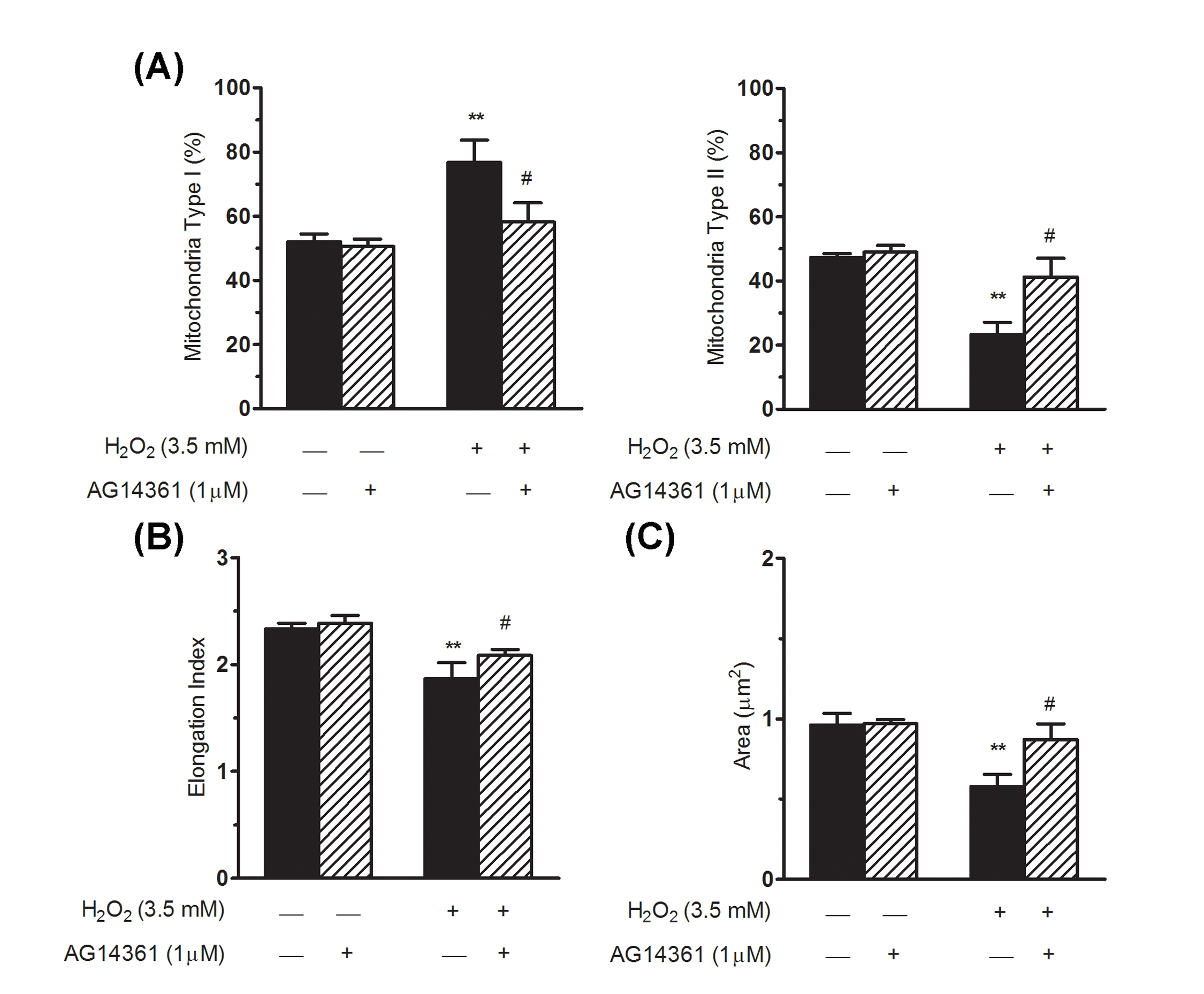

Supplement: S4 Fig — (A) Percentage of type I (small globular; left graph) and type II (tubular including linear, twisted, branched and looped forms; right graph) of mitochondrial morphology prior to quantifying mitochondrial morphology with MicroP software. WRL68 cells pre-incubated 16 h with AG14361 were treated with H2O2 for 30 min and then post-incubated for 4 h. (B) Elongation index of mitochondria. (C) Area of mitochondria. At least 7000 mitochondria were analysed with MicroP software in each condition and in each experiment. Bars represent the mean ± SEM of three independent experiments. Significant differences: **P < 0.01 with respect to the control (non-treated cells); #P < 0.05 with respect to 3.5 mM H2O2. (TIF) [file pone.0187130.s004.tif]
